# Coordination chemistry suggests that independently observed benefits of metformin and Zn^2+^ against COVID-19 are not independent

**DOI:** 10.1007/s10534-024-00590-5

**Published:** 2024-04-05

**Authors:** Thomas D. Lockwood

**Affiliations:** Department Pharmacology and Toxicology, School of Medicine, Wright State University, Dayton, OH 45435 USA

**Keywords:** Metformin, Zn^2+^, COVID-19 outcomes, Metformin–Zn^2+^ complex, Zn^2+^-moderated protease web, Inflammatory cardiomyopathy

## Abstract

Independent trials indicate that either oral Zn^2+^ or metformin can separately improve COVID-19 outcomes by approximately 40%. Coordination chemistry predicts a mechanistic relationship and therapeutic synergy. Zn^2+^ deficit is a known risk factor for both COVID-19 and non-infectious inflammation. Most dietary Zn^2+^ is not absorbed. Metformin is a naked ligand that presumably increases intestinal Zn^2+^ bioavailability and active absorption by cation transporters known to transport metformin. Intracellular Zn^2+^ provides a natural buffer of many protease reactions; the variable “set point” is determined by Zn^2+^ regulation or availability. A Zn^2+^-interactive protease network is suggested here. The two viral cysteine proteases are therapeutic targets against COVID-19. Viral and many host proteases are submaximally inhibited by exchangeable cell Zn^2+^. Inhibition of cysteine proteases can improve COVID-19 outcomes and non-infectious inflammation. Metformin reportedly enhances the natural moderating effect of Zn^2+^ on bioassayed proteome degradation. Firstly, the dissociable metformin–Zn^2+^ complex could be actively transported by intestinal cation transporters; thereby creating artificial pathways of absorption and increased body Zn^2+^ content. Secondly, metformin Zn^2+^ coordination can create a non-natural protease inhibitor independent of cell Zn^2+^ content. Moderation of peptidolytic reactions by either or both mechanisms could slow (a) viral multiplication (b) viral invasion and (c) the pathogenic host inflammatory response. These combined actions could allow development of acquired immunity to clear the infection before life-threatening inflammation. Nirmatrelvir (Paxlovid®) opposes COVID-19 by selective inhibition the viral main protease by a Zn^2+^-independent mechanism. Pending safety evaluation, predictable synergistic benefits of metformin and Zn^2+^, and perhaps metformin/Zn^2+^/Paxlovid® co-administration should be investigated.

## Introduction

The tragedy of COVID-19 has focused attention on three seemingly unrelated topics: (a) viral and host proteases, (b) Zn^2+^, and (c) the amazing “polytherapeutic” effects of metformin against infectious and non-infectious diseases. Integration of familiar facts provides an unfamiliar conclusion. Consideration of coordination chemistry, virology, protease enzymology, and the pathobiology of inflammation suggests an unappreciated relationship between these topics. Confluence of a large amount disparate evidence is reviewed here; however, many questions remain.

**Table 1:** Some of the Zn^2+^—responsive Intracellular Proteases

Proteases Inhibited by Zn^2+^ or Zn^2+^—binding agents	Catalytic mechanism	Mechanism of Zn^2+^ inhibition	Illustrative references among others
SARS-CoV-2 Main Protease SARS-CoV-2 Papain-like Protease	Cysteine	Inhibition of catalytic mechanisms (Function of PLPro Zn^2+^-binding site is unknown)	Barretto et al. (2005), Lee et al (2009), Liu (2015), AliáKhan (2021), DeLaney et al (2021), Grifagni et al (2021), Kladnik et al (2022), Shetler et al (2022)
Cysteine Cathepsins, e.g. Cathepsin B	Cysteine	Inhibition of catalytic mechanism, Others?	Reviewed in Lockwood (2010, 2013, 2019)
Calpains	Cysteine	Inhibition of catalytic mechanism and Interaction with E-F hand structure	Myoshi et al. (2016).
Caspases	Cysteine	Inhibition of catalytic mechanism and Binding to allosteric exosites	Perry et al. (1997), Eron et al. (2018)
Furin	Serine	Mixed	Podsiadlo et al. (2004)
Some of the Kallikreins	Serine	Inhibitions of catalytic Mechanisms, Various alterations of higher-order structures	Debela et al. (2018) and other papers from this group
Proteasome-associated Deubiquitinases (DUBs) -Ubiquitin-specific protease 14 (USP14) -Ubiquitin carboxyl terminal hydrolase L5 (UCHL5)	Cysteine	Inhibition of catalytic mechanism of DUBs by Zn^2+^ causes intracellular accumulation of ubiquitin-conjugated proteasome substrates	Kim (2004), Liu et al. (2015), Chen et al. (2017), Zhao et al (2017)
26S proteasome holoenzyme activity	Threonine	Influences on assembly state or reaction mechanism or conformation (work in progress)	Kiss et al. (2005), Chouduri et al (2008) Cvek et al. (2008), Frezza (2009), Verani et al. (2012), Ahmad et al. (2020) and others

**Fig. 1:**
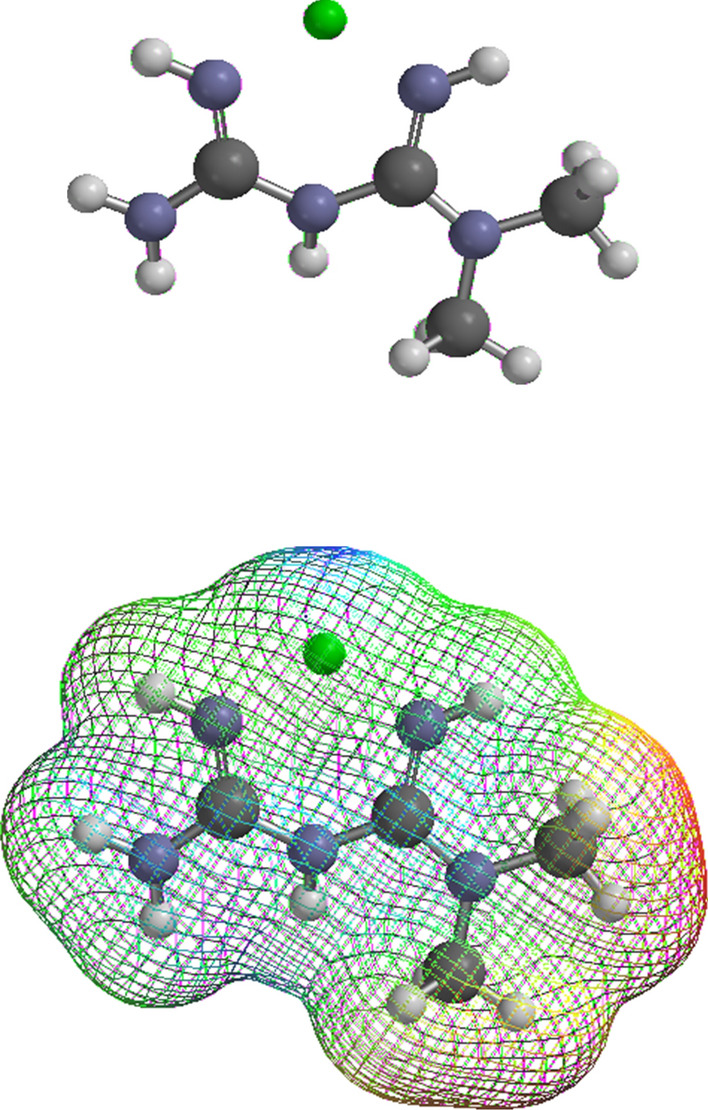
The metformin–Zn^2+^ complex computed as “in vacuo”. The structure of the Zn^2+^ complex of metformin was computed with the Spartan’08 program (Wavefunction, Inc., Irvine, CA) using density functional theory at the DFT B3LYP/6-31G* level. Zn^2+^ forms a 1:1 bidentate complex through the lone electron pairs of imino nitrogens at the 2 and 4 positions of biguanide. Experimental evidence indicates that the 2 and 4 nitrogen positions remain protonated as computed. The charge on the complex is 2+; however, electron density is transferred from metformin to Zn^2+^. Counterions of a biological mixture are not shown. The metformin complex leaves unoccupied ligand-binding sites of Zn^2+^ exposed to interactions with additional ligands. Many metabolites and macromolecules can undergo ligand exchange with the dissociable metformin–Zn^2+^ (see text). Metformin can form a mixed (heteroleptic) complex with an endogenous biomolecule coordinated around the central metal cation. Zn^2+^ is shown in green, carbon: black, nitrogen: blue, hydrogen: white. The surface potential gradation in the mesh is as indicated in Fig. 2

**Fig. 2:**
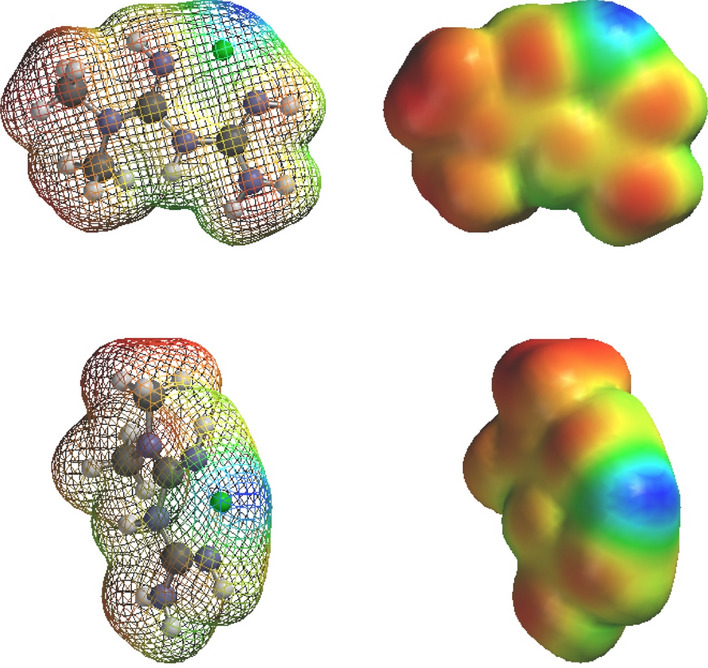
Surface potential gradient of the metformin–Zn^2+^ complex. The mesh is shown as a solid surface with the potential gradient color coded from more negative to more positive by red, orange, yellow, green, blue. Transfer of electron density from the metformin ligand to the Zn^2+^ cation masks much of the exposed positivity of Zn^2+^ as illustrated by the small blue area. Metformin can acquire dietary Zn^2+^ bound to foodstuffs in the gut thereby increasing its low bioavailability. Metformin and presumably its Zn^2+^ complexes can be actively co-transported from the gut and between tissues by various cation transporters (see text). Thus, metformin can serve as a Zn^2+^ carrier (“zincophore”) increasing bioavailability and creating new pathways of active Zn^2+^ translocation. Firstly, the metformin–metal complex is dissociable following absorption and might increase body Zn^2+^ content. Secondly, some biguanide Zn^2+^ complexes are demonstrated to be non-natural protease inhibitors independent of any change in the amount of body Zn^2+^. Mixed complexes can form with metformin sharing a central Zn^2+^ with the thiolate-imidazole catalytic partners of viral and host cysteine proteases (Lockwood 2019)

**Fig. 3:**
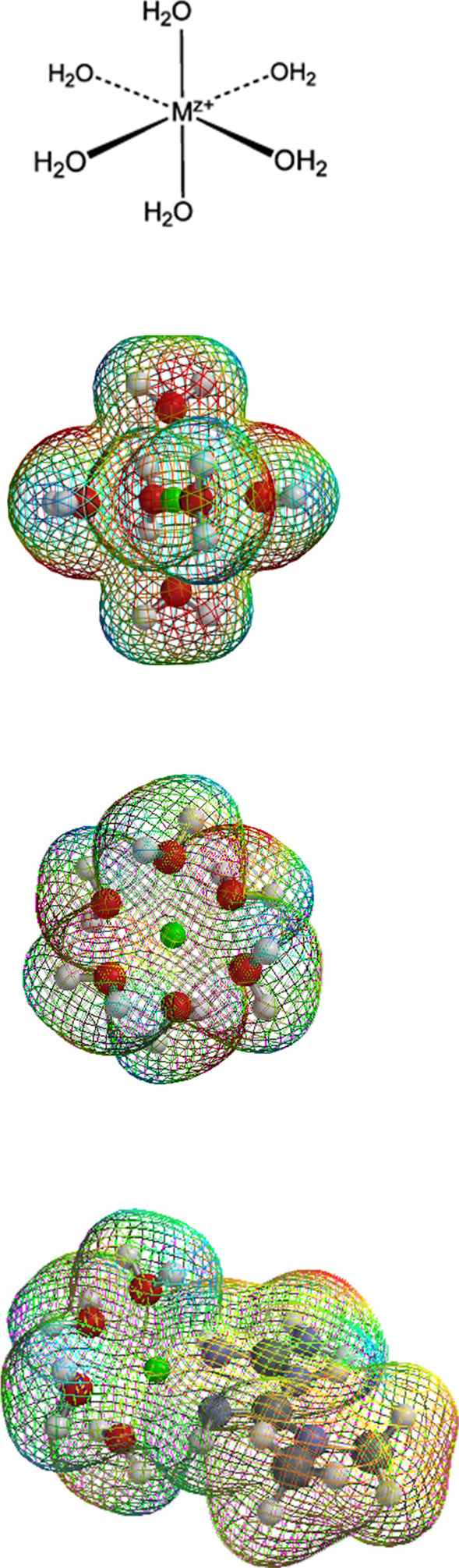
Solvation of Zn^2+^ and the metformin–Zn^2+^ complex. As with many other metals, solvated Zn^2+^ has six hydrogen-bonded water molecules in the first hydration shell (see text). The vertices of the ligand-binding sites define an octahedron. Two perspectives of the hydrogen-bonded waters are illustrated. Firstly, bidentate coordination of Zn^2+^ by the two imino nitrogens of metformin can displace two of the waters and decrease solvent attraction. Secondly the methyl groups of metformin create hydrophobicity and promote hydrophobic binding. In the absence of metformin, solvated inhibitory Zn^2+^ can bind to the thiolate-imidazole catalytic partners of a protease with concentration dependence. Firstly, metformin can increase the natural inhibitory binding of Zn^2+^ to the thiolate-imidazole catalytic partners merely by decreasing solvent attraction for Zn^2+^. Secondly, the methyl groups of metformin can act as a bridge between Zn^2+^ and subsites surrounding the catalytic partners; this can increase the stability of the association and fraction of time that inhibitory Zn^2+^ is interactive with the reaction. Thirdly, other biguanide derivatives with certain structural features can competitively inhibit substrate binding to subsites independent of Zn^2+^, e.g. phenformin. A “Zn^2+^ sandwich” can form between various biguanide derivatives and the catalytic partners of a protease (illustrated with phenformin in Sweeney et al 2003). Inhibition of a peptidolytic reaction is proportional to the concentration-dependent stability of the [protease–Zn^2+^–metformin] association. By these combined mechanisms, various biguanide derivatives can enhance the natural potency and inhibitory actions of Zn^2+^ toward protease reactions

**Fig. 4:**
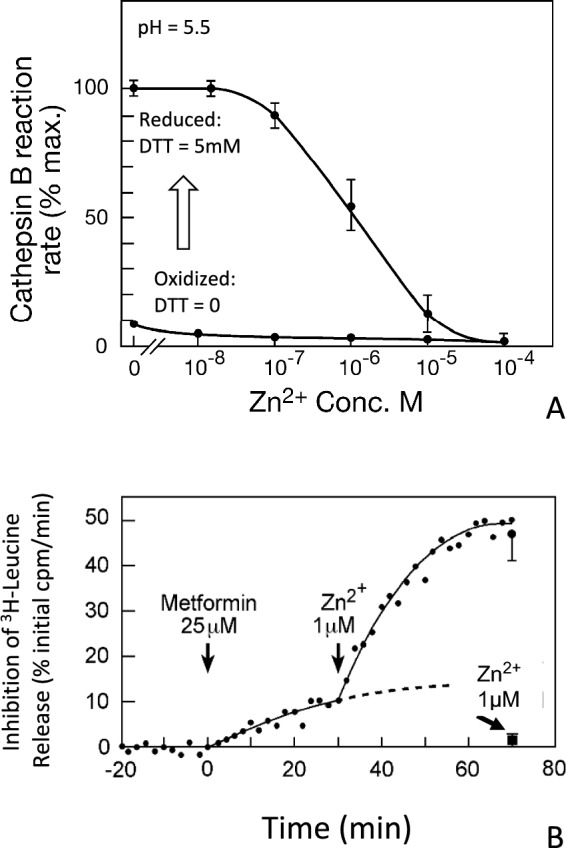
**A** Responsiveness of maximally reduced cathepsin B reaction rate to Zn^2+^ concentration (reproduced from Lockwood 2010). Inactivated (air-oxidized) cathepsin B and Zn^2+^ concentrations were added to buffer (pH 5.5) and saturating substrate concentration (25 µM CBZ-Phe-Arg-AMC) and assayed without reductive activator (bottom trace). DTT (5 mM) was then added; and the reactions were again measured after reductive re-activation. The 100-fold inhibitory concentration range of Zn^2+^ from approximately 0.1 to 10 µM (EC-50 = approximately 1 µM) is consistent with moderation of intracellular activity by the range of exchangeable intracellular Zn^2+^ concentrations; however, conditions are not directly comparable (see text). **B** The inhibitory effect of metformin on myocardial proteome degradation in the absence of extracellular Zn^2+^, and the synergistic effect of Zn^2+^ on metformin action (reproduced from Lockwood 2010). Proteins of the perfused rat heart were biosynthetically labelled with ^3^H leucine. The minute-to-minute rate of leucine release was measured in the non-recirculating effluent perfusate (see text). Experimental agents were delivered into the flowing perfusate at a constant rate from 100-fold concentrated solutions by an infusion pump. In the absence of metformin, basal protein degradation over this observation time was the same with and without sub-physiological 1µM extracellular Zn^2+^ (see text). Sustained infusion of therapeutic metformin concentration alone (25 µM) caused 10–15% inhibition in the artificial absence of extracellular Zn^2+^. However, when sub-physiological 1 µM Zn^2+^ infusion was added to prior metformin infusion an immediate synergy resulted (see text). Addition of higher Zn^2+^ concentration caused greater inhibition

## Separate human trials reveal that COVID-19 outcomes can be improved by treatment with either oral Zn^2+^ supplementation or metformin

These separate investigations were prospective trials comparing randomized treated and control populations. The chemical and biological bases of these phenomena are unexplained; and no connection between them has been proposed. In one study, the outcomes of COVID-19 were improved by Zn^2+^ at 50 mg/day beginning shortly after infection:

… “Oral zinc treatment for 15 days is associated with a nearly 40% reduction in death and ICU admission, with shortening of symptom duration in patients with COVID-19. Our results have very important clinical relevance in the absence of specific effective curative treatment”... (Ben Abdallah et al. 2023).

With regard to this report, an editorial opinion from the New England Journal of Medicine offered a reasonable suggestion:

… “for patients or clinicians who wish to try zinc in addition to other recommended treatments, there's probably little downside — and now some scientific justification ”… (Kaul 2022, and see Vinceti et al. 2023).

Separate trials found that COVID-19 outcomes were improved by initiation of metformin, beginning shortly after infection:

… “Metformin had a 42% reduction in ER visits/hospitalizations/death through 14 days; a 58% reduction in hospitalizations/death through 28 days, and a 42% reduction in Long COVID through 10 months”… (Bramonte et al. 2023; Erickson 2023; Boulware 2023).

Many retrospective studies had previously indicated that pre-infection administration of metformin improved COVID-19 outcomes in diabetic individuals (Ibrahim 2021; Lalau et al. 2021; Chan et al. 2022; Wiernsperger et al. 2022; Ma et al. 2023; Pedrosa et al. 2023; Yen et al. 2023 and others). Prior metformin administration also improved COVID-19 in non-diabetics taking it for polycystic ovary syndrome, and pre-diabetics taking it for preventive risk reduction (Chan et al. 2022). These unexplained benefits are not due to the anti-hyperglycemic action of metformin.

Although metformin and Zn^2+^ independently improve COVID-19 outcomes by 40%, their combined effect is unknown. Metformin and oral Zn^2+^ also have separate actions against an amazing variety of non-infectious diseases caused by unrelated primary etiologies. The continuing saga of this amazing drug is far from completed.

## Chemical and biological background suggests a connection between the separate benefits of metformin and Zn^2+^ supplementation against COVID-19 and non-infectious diseases

Morbidity and mortality from COVID-19 are associated with the host inflammatory response to the increasing viral load (Diamond and Kanneganti 2022; Paludan and Mogensen 2022; Wang et al. 2023; Yamada and Takaoka 2023).

Inflammation (“to set on fire”) is a ridiculous term referring to much of innate immunity. Acute and chronic inflammation are undefined concepts that are used here advisedly. Inflammation from infectious and non-infectious causes have much in common. COVID-19 involves virally encoded protease activities involved in multiplication/invasion; however, host proteases execute the pathogenic inflammatory response. “Long COVID” results from continuation of residual inflammation and auto-immunity after the virus has been cleared (Gasecka et al. 2021; Sidik 2022; Xie et al. 2022; Sewanan et al. 2023). The complexities of low-grade and high-grade inflammation can also contribute to diverse non-infectious disease etiologies. Acute and chronic inflammatory processes involve a network of many proteases in diverse cell types (see Table 1). Indeed, many human proteases might play a role in the graded spectrum from reversible hyper-catabolism to programmed forms of cell death.

A common trait shared by the two viral proteases and many host proteases has been overlooked. The reaction rates of many (but not all) proteases are naturally submaximal under the range of exchangeable biological Zn^2+^ concentrations (Table 1). However, Zn^2+^ has not been mentioned among factors controlling proteases (López-Otín and Bond 2008). Evidence suggests that separate benefits of metformin and Zn^2+^ against COVID-19 are associated with enhancement of the natural moderating effect of Zn^2+^ on viral and host protease activities. It has long been known that biguanide derivatives form coordination complexes with Zn^2+^ and other metals (extensively reviewed by Rusanov et al. 2022). Computational chemistry provides additional insights (Fig. 1). Exchangeable cell Zn^2+^ concentration naturally moderates (a) viral protease reactions required for multiplication, (b) hijacked human protease reactions required for viral invasion and (c) human protease reactions involved the pathogenic inflammatory response. Zn^2+^ is the only known biological factor that simultaneously decreases all viral and host protease activities of Table 1 and more.

## It is suggested here that metformin coordination of Zn^2+^ decreases the activities of virally encoded proteases and many host proteases by two conceivable mechanisms

Zn^2+^ can be transported as the independent cation or co-transported as bound to many other substances (Haydon and Cobbett 2007; Kocyła et al. 2021; Stanton et al. 2022). Firstly, the dissociable metformin complex could serve as a Zn^2+^ carrier that increases the active intestinal absorption, inter-organ transport, distribution and perhaps body content of naturally inhibitory, independent Zn^2+^. Secondly, the metformin–Zn^2+^ complex could be a non-natural direct inhibitor of diverse proteases without changing exchangeable Zn^2+^ concentrations. Some combination of both mechanisms is probably operative. Simultaneous suppression of viral multiplication and the pathogenic inflammatory response provides time for acquired immunity to arise and clear the infection before a cytokine storm; this allows a largely symptom-free course of disease.

The anti-proteolytic hypothesis of metformin–Zn^2+^ interactions against inflammation does not eliminate additional mechanisms, including Zn^2+^-independent mechanisms (not reviewed here). The many roles of Zn^2+^ include signaling networks, transcription, molecular structure, mitochondrial function, autophagy and hundreds of enzyme reactions other than proteases (e.g. Te Velthuis et al. 2010; Thompson 2022; Costa et al. 2023). Metformin coordination might modify the influences of Zn^2+^ on kinase and phosphatase signaling. Zn^2+^ is known to be involved in protein–DNA interactions and protein–protein interactions (Kocyła et al 2021). Zn^2+^ can form a natural bridge between the surface of proteins, acting as a “glue” between them (Lu 2010). Conformational change or unfolding of proteins can influence their susceptibility to degradation. Binding of pharmacological metformin–metal complexes to surface Zn^2+^ could modify macromolecular conformations and functions of some proteins, e.g. transcriptional regulatory proteins such as P53 (Ha et al. 2022). Indeed, Zn^2+^ is biologically pervasive with many functions that metformin might modify.

## Why do some individuals experience no symptoms of SARS-CoV-2 infections, while others develop a lethal inflammatory response?

Many investigators have reported that prior Zn^2+^ deficiency is a risk factor for COVID-19 susceptibility and/or outcomes (Jothimani et al. 2020; Wessels et al. 2020, 2022; Joachimiak 2021; Vogel-González 2021; Maares et al. 2022; Olczak-Pruc et al. 2022; Tabatabaeizadeh et al 2022; Almasaud et al. 2023; Rheingold et al. 2023). Inadequate Zn^2+^ might allow a combination of increased viral multiplication, viral invasion and pathogenic host inflammation. A spectrum of Zn^2+^ deficiencies can result from a combination of genetic and acquired causes. Body metal content results from the relative rates of input from the gut vs. output by all excretory routes; both processes are under transcriptional control of many proteins involved. Individual variability in body Zn^2+^ content and distribution can be caused by differences in dietary intake, gastrointestinal surgery, bowel inflammation, common drugs that decrease Zn^2+^ absorption or increase excretion, genetic variation Zn^2+^ regulation and other factors. Exchangeable body Zn^2+^ content can fluctuate on a weekly basis. Zn^2+^ adequacy protects against infectious and non-infectious inflammation of the lung and other tissues (Skalny et al 2020); Zn^2+^ inadequacy increases inflammation. The total body Zn^2+^ content optimizing health and longevity is uncertain. Zn^2+^ supplementation might have both anti-viral and anti-inflammatory actions by the four anti-proteolytic mechanisms listed below.

Heritable or acquired Zn^2+^ deficit can be severely inflammatory under non-infectious conditions. Recent studies indicate that body Zn^2+^ deficit can cause or contribute to damage of skin, heart and other tissues in the absence of infection (Kodama et al 2020; Skalny et al 2020; Rosenblum et al 2020; Zou et al 2023, and additional citations below). In most cases timely Zn^2+^ supplementation can slow, stop or sometimes reverse sterile inflammation associated with Zn^2+^ deficiency. The exact relationship between plasma Zn^2+^ measurements and “whole body” Zn^2+^ content is uncertain. It has been estimated that 17% of the world population has a medically significant dietary Zn^2+^ deficit (Wessells and Brown 2012). An unknown fraction of the population in developed regions might have a body Zn^2+^ content that is inadequate for optimal health and longevity.

Genomics has not yet accounted for all of the many proteins involved in systemic, cellular and subcellular Zn^2+^ input vs. output in various compartments. Zn^2+^ transport and electrophysiology are incompletely understood. Zn^2+^ regulation includes transporters and channels that accept the independent cation (Kambe et al 2021; Hara et al 2022), and co-transporters that translocate Zn^2+^ that is bound to many other substances. In brief, the human genome encodes more than 400 transporters in superfamilies. The ABC transporters utilize ATP to transport substrates across the membrane against their concentration gradients. SLCs include symporters and anti-porters coupling transport of one substance to the gradient of another. Ion channels pass Zn^2+^ down its concentration gradient. Reviews emphasize the 10 ZnT (SLC30A1-10) and 14 ZIP (SLC39A1-14) Zn^2+^ transporters operating across membranes in opposite directions*.* However, multiple organic cation transporters (OCTs), multidrug and toxicant transporters (MATEs) and ABC transporters accept Zn^2+^-binding substances including metformin (see below). In addition, the exchangeable Zn^2+^ concentration in various compartments depends upon the enormous Zn^2+^ interactome of metabolites and macromolecules ranging from citrate to metallothionein. The many proteins involved in transport and binding of Zn^2+^ suggest much genetic variation in the regulation of its exchangeable concentration.

## Insights into the treatment of COVID-19 with metformin can be gained from the treatment of an inflammatory disease known as acrodermatitis enteropathica with a drug named diodoquin

In the early 1940s dermatologists described a disease of unexplained etiology involving life-threatening inflammation and disintegration of skin [history reviewed by Khan et al (2015)]. It was originally suspected that acrodermatitis enteropathica (AE) was an infectious disease; however, no evidence of microbes could be found.

Years later it was reported that this disease could be successfully treated with a drug being used for treatment of intestinal infection with amoeba. However, the inflammation returned when diodoquine (diiodohydroxyquinoline) was discontinued. It was later discovered that AE was dramatically “cured” beginning within days of oral Zn^2+^ supplementation. The mystery was solved in the 1970s when it was found that diodoquine is a Zn^2+^ -binding substance that treats AE by increasing the defective absorption of Zn^2+^ and perhaps systemic compartmental kinetics (reviewed by Agget et al. 1979).

AE is now known to be an autosomal recessive inflammatory condition caused by intestinal malabsorption of Zn^2+^. AE is still referred to as an inflammatory disease of skin because dermatologists can see it there; however, other organs are involved, e.g. the heart (Rosenblum et al. 2020; Ozyildirim and Baltaci 2023). Indeed, AE is a single name for a disease spectrum. The severity, time course and pattern of skin lesions ranges from a lethal syndrome shortly after birth to chronic inflammatory lesions in various locations of the adult body. Most intestinal Zn^2+^ absorption is mediated by the ZIP4 transporter encoded by the SLC39A4 gene. More than 50 genetic mistakes in the SLC39A4 gene have been linked to Zn^2+^ deficiency, including regulatory mutations, nonsense mutations, missense mutations, frameshift mutations, splice site mutations, etc. (Kuliyev et al. 2021 and others). However, genetic variation in additional Zn^2+^ regulatory proteins is likely. Insufficient Zn^2+^ intake and/or the spectrum of dysregulations could be hidden contributors to non-infectious low-grade inflammatory conditions including heart diseases (see below).

Diodoquine reverses the inflammation of AE by creating an artificial pathway(s) of intestinal Zn^2+^ transport which bypasses the defective transporter. Diodoquine was an early example of Zn^2+^—carrying drugs (Scavo and Oliveri 2022). Pharmacological “zincophores” can also compete with the large number of dietary Zn^2+^—binding substances in the gut (Hall and King 2023). Zn^2+^-binding xenobiotics can enhance intestinal Zn^2+^ bioavailability by removing it from foodstuff. In addition, active absorption of Zn^2+^-binding xenobiotics can co-transport their bound Zn^2+^ (Krenn et al. 2009 and see below). Conversely, Zn^2+^-binding foodstuffs or drugs that are not absorbable are known to cause Zn^2+^ deficiency by carrying intestinal Zn^2+^ out of the body. Following absorption, Zn^2+^ carriers can also modify the natural kinetics, distribution and excretion of Zn^2+^. However, diodoquine and some other Zn^2+^ carriers might directly inhibit proteases as well as co-transporting Zn^2+^ (Yuan et al. 2020b; Shahabadi et al. 2021; Scavo and Oliveri 2022, and Table 1). Metformin forms dissociable coordination complexes with metal cations in the gut (Figs. 1-3). There is no reason to doubt that active intestinal absorption of metformin by multiple cation transporters can also co-transport bound Zn^2+^ and other metals (citations below). However, certain biguanides have been shown to be direct protease inhibitors (Lockwood 2019). The question of whether the metformin–Zn^2+^ complex functions primarily as a dissociable Zn^2+^ carrier or a direct protease inhibitor is a major shortcoming of the present hypothesis; this observer suspects that both mechanisms are in operation (see below).

## Inhibition of viral and host proteases are effective mechanisms against COVID-19 and non-infectious inflammation

The SARS-CoV-2 virus life cycle requires multiple proteolytic events mediated by the two virally encoded cysteine proteases and several host proteases identified thus far (Klemm et al 2020; Maiti et al 2020; Wu et al 2020; Lee et al 2022; Müller et al 2022; Oubahmane et al 2022; Pizzato et al 2022; Farkaš et al 2023, Li et al 2023). In addition, excessive activities of host proteases of Table 1 are involved in the inflammatory response to infectious and non-infectious stimuli. The main viral protease serves to process the viral polyprotein with high specificity and selectivity. The papain-like viral protease is required for viral maturation (Han et al 2005); however, its multiple functions are uncertain. The papain-like protease has broad substrate acceptance, including possible de-ubiquitinating activity of unknown significance (Barretto et al 2005; Lindner et al 2005).

Degradation of 20,000 gene products by the “degradome” is mediated by only five peptidolytic reaction mechanisms. Various proteases have a wide variety of substrate preferences. However, substrate binding and the catalytic reaction mechanism are separate steps in protease function. Inhibition of a single peptidolytic reaction mechanism can decrease the action of many proteases on many proteins independent of substrate preference. One of these reaction mechanisms is quite sensitive to relevant Zn^2+^ concentrations (cysteine proteases), and another is moderately sensitive (serine proteases) (Lockwood 2019). In contrast, carboxyl and metalloprotease mechanisms are completely Zn^2+^-insensitive. The relative Zn^2+^ sensitivity of threonine proteases reactions are uncertain. However, Nature can engineer Zn^2+^ sensitivity of any protease by its effect on higher-order structure independent of the reaction mechanism, e.g. allosteric exosites or subunit assembly state (Eron et al. 2018 and Table 1).

Effective substrate-binding aligns a scissile peptide bond with the catalytic dyad or triad long enough for a hydrolytic reaction to occur. Substrate binding to the protease does not mediate peptide hydrolysis by the catalytic partners. Conversely, the catalytic reaction mechanism does not determine substrate binding specificity or affinity. Interference with the peptidolytic reaction mechanism can be independent of substrate binding and vice versa. Accordingly, proteases can be pharmacologically modulated by interference with substrate binding or the catalytic reaction mechanism or both simultaneously. In theory, Zn^2+^ alone or a Zn^2+^-interactive drug can simultaneously decrease the degradation of many proteins by many proteases independent of their substrate specificities or preferences. Pharmacological intervention in the peptidolytic reaction mechanism has received little attention; however, Nature employs this control.

Both of the virally encoded proteases are cysteine proteases with the primordial Cys(thiolate)-His(imidazole) catalytic partners shared by all cysteine proteases. Various combinations of nCys neighboring nHis have peculiar multidentate affinity for Zn^2+^ in many “Zn^2+^ finger” protein binding sites. Biological Zn^2+^ fluctuations can simultaneously influence the reaction rates of both viral CysHis proteases and diverse host CysHis proteases despite very different substrate specificities and functions.

Pfizer’s Paxlovid® is a successful viral protease inhibitor, and a tribute to the discovery and development teams. Paxlovid® acts by specific covalent binding to the viral main protease independent of Zn^2+^. The viral main protease has no counterpart among human proteases. Paxlovid® combines a specific, selective, covalent inhibitor of only the viral main protease (nirmatrelvir) and an inhibitor of its metabolism (ritonavir) (Owen et al 2021; Marzi et al 2022). The substrate specificity of the viral main protease differs from all human proteases; and nirmatrelvir does not inhibit host proteases. In contrast, the effects of Zn^2+^ on viral and diverse host protease activities differ markedly from nirmatrelvir. At least four effects of Zn^2+^ on COVID-19 can be distinguished:

1. Zn^2+^ can decrease the two viral cysteine protease reactions required for multiplication and invasion as reported in many ex vivo studies (reviewed by Lee 2009; do Nascimento et al. 2022; Kladnick et al. 2022; Li et al. 2023a; Shetler et al. 2022 and others).

2. Zn^2+^ can also decrease host protease reactions that are hijacked for the viral life cycle, e.g. furin or cathepsins L or B (Hashimoto et al 2021; Shirbhate et al 2021; Zhao et al 2021; Ding et al 2022; Huang et al 2022, Table 1 and others).

3. Zn^2+^ can decrease many additional host protease reactions that execute the inflammatory response to infectious or non-infectious causes (El‐Shimy et al 2021; Rahbar Saadat et al 2021; Lv et al 2022, Table 1 and others).

4. Zn^2+^-responsive degradative control might involve more than its effect on protease reactions. In addition to inhibiting proteases, Zn^2+^ suppresses autophagy at least in some cell types. The effect of Zn^2+^ on the integrity of mammalian cell membranes is largely uncharacterized. Multiple studies agree that Zn^2+^ starvation elevates access of substrates to vacuolar proteases (Richie and Askew 2008; Eguchi et al. 2017; Horie et al. 2017; Kawamata et al. 2017; Ding and Zhong 2017; Gross and Graef 2020; Zlobin 2021; Kim et al. 2022b and others). Autophagy has been suspected of a role in COVID-19 (He et al. 2022; Ivanova et al. 2023).

Selective inhibition of the viral main protease by Paxlovid® via a Zn^2+^-independent mechanism resulted in an improvement of 80% in COVID-19 outcomes (Reis et al. 2022, Lewnard et al. 2023). Although Paxlovid® is a strong inhibitor, its action in all body compartments is presumably incomplete. The independent benefits of Zn^2+^ or metformin are each near 40% (reviewed above). A supratherapeutic amount of Zn^2+^ can completely stop any cysteine protease reaction ex vivo, including both of the vial proteases; however, this amount of Zn^2+^ is not safely attainable in vivo. In theory, submaximal inhibition of the two viral proteases and many host proteases by relevant Zn^2+^ concentrations might have a submaximal therapeutic action on both viral multiplication/invasion and the pathogenic host inflammatory response. Metformin might cause synergistic enhancement of the natural action of Zn^2+^ against viral and host proteases. Simultaneous administration of metformin, Zn^2+^ and Paxlovid® might safely provide a greater therapeutic advantage.

Emergence of resistance to initially effective protease inhibitors is a major problem (Robert 2022). Mutations in the primary structure of a protease can lead to decreased inhibitor binding within and around the active site. The reaction mechanisms of all cysteine and serine proteases require formation of an ion pair between cysteine (thiolate) or serine (alkoxide) and the imidazole (imidazolium) ring of the nearby histidine partner. Zn^2+^ inhibits by insertion between thiolate and imidazole thereby preventing proton transfer and formation of the ion pair. Mutation to Zn^2+^ insensitivity of a protease reaction is much the same as mutation to a loss of the reaction mechanism. Mutation to a completely Zn^2+^-resistant cysteine protease that retains function is unlikely.

## Science has not yet appreciated Nature’s control of the reaction rates of a proteases network by Zn^2+^

It is speculated that alternative splicing of 20,000 human gene products might produce a half million members of the proteome; this results in a multitude of different cleavage sites for proteases. The genome reportedly encodes 273 extracellular proteases, 277 intracellular proteases, and 16 integral membrane proteases (Overall and Blobel 2007). These hydrolases evolved from only five primordial peptidolytic reaction mechanisms (Klein et al. 2018). Coordination of simultaneous changes in hydrolysis of nearly a half million proteins  by 277 proteases has remained a fundamental mystery. Bulk proteome turnover can be influenced by such factors as changes in expression of protease content and/or specificity (Zhou et al. 2020), and/or expression of approximately 200 proteinaceous protease inhibitors (Puente and López-Otín 2004; Rawlings 2010; Tušar et al. 2021), and/or diverse post-translational modifications of substrate susceptibility to proteases, and/or autophagy, and/or membrane/cytoskeletal properties etc. However, the integrated control of proteome turnover cannot be entirely explained by such factors.

Zn^2+^ appears to be a primordial “pacemaker” of diverse protease reactions sharing cellular compartments and catalytic environments. Coordinated modulation of protease reaction mechanisms by Zn^2+^ has been largely ignored. It has not been appreciated that the Zn^2+^-responsive proteases of Table 1 comprise most of the 277 intracellular proteases; however many questions arise. The exact hierarchy of Zn^2+^ sensitivities is not precisely known. Most of the Zn^2+^-responsive protease reaction rates are submaximal under biological concentrations of exchangeable Zn^2+^; however, some might be completely inactivated. Zn^2+^ modulation of some protease reaction rates includes interactions with both catalytic reactions and allosteric exosites (Table 1 and see below). Compartmental regulation of Zn^2+^ might be among influences on both lysosomal and extra-lysosomal pathways including proteasomal degradation. Lysosomal proteases have diverse functions outside the lysosome and cell (Xie et al 2023). However, other metals might also be players. The interaction of Zn^2+^ with transcriptional regulation of the ratio of proteases and proteinaceous protease inhibitors is uncharacterized. Each cell type might differ. Zn^2+^ deficiency elevates autophagy in some cell types (citations above). In theory, control of exchangeable cell Zn^2+^ can simultaneously impose changes in the reaction rates of viral and host proteases sharing the same catalytic environments.

Inhibition of cysteine protease assays by endogenous metal cations has been known since the 1920s. Biological and non-biological metals have a range of inhibitory potencies on various proteases (Novinec et al. 2022). At the beginning of this millennium it was discovered that Zn^2+^ suppresses apoptosis (Perry et al. 1997; Chai et al. 1999) and tissue protein degradation (Sweeney et al. 2003). However, it has not been recognized that intracellular proteases serving very different functions share a common responsiveness to intracellular Zn^2+^. The Zn^2+^-responsive proteases listed in Table 1 have been implicated in controlled and uncontrolled inflammation in the absence of viruses.

Importantly, exchangeable biological Zn^2+^ concentrations do not inhibit most proteases completely. Most of the protease reactions in the Zn^2+^-responsive web are submaximal under biological Zn^2+^ but not stopped. Indeed, excessive inhibition of diverse host proteases can be pathogenic. The exchangeable cell Zn^2+^ concentration range is a moderator of most protease reactions rather than an inhibitor. However, the effects of Zn^2+^ on higher-order structure of some proteases has introduced a range of Zn^2+^ responsiveness. Some of the caspases might be completely or almost completely inactivated under basal cell Zn^2+^ (Eron et al. 2018). The E–F hand structure (calmodulin loop) responds to both Zn^2+^ and Ca^2+^ (Kozlyuk et al. 2019). The reaction mechanism of calpains is sensitive to Zn^2+^; however, Zn^2+^ responsiveness of their calmodulin loop is uncertain. It appears that the complexities of mammalian proteome turnover evolved under a primordial buffer consisting of metals, redox and protons (Lockwood 2010); and this buffer is regulated by the cell. A graded hierarchy of Zn^2+^ sensitivities might permit complete inhibition of some proteases while allowing the reactions and functions of others.

In short: Zn^2+^ is a major component of an endogenous protease reaction buffer with a variable “set point”. Zn^2+^ acts as a “pan-protease” modifier that inactivates some protease activities and maintains many others at submaximal reaction rates if body Zn^2+^ content is adequate. Decreasing concentration of exchangeable Zn^2+^ increases the ongoing activities of many proteases and can recruit inactive protease zymogens. Extreme Zn^2+^ deficit weakens the buffer and unleashes reversible hyper-catabolism or programed death (Perry et al. 1997; Chai et al 1999).

The severity of COVID-19 depends upon the viral load and the pathogenic host inflammatory response to the viral load (Diamond and Kanneganti 2022; Paludan and Mogensen 2022; Wang et al. 2023; Yamada and Takaoka 2023).

Current therapy includes anti-viral agents and the anti-inflammatory steroid, dexamethasone. In theory, protease moderation with Zn^2+^-interactive agents might target both virus life cycle and host inflammatory response simultaneously. Partial suppression of viral and host proteases does not cure the infection. “Whole body” inhibition of viral proteases and certain host proteases can slow viral multiplication and invasion and simultaneously decrease or delay the host inflammatory response. *In COVID-19, “whole body” moderation of the Zn*^*2+*^*-interactive web of host protease activities by metformin can decrease innate immunity more than acquired immunity.* Simultaneous decrease in viral and host protease activities provides time for development of immunity to clear the infection before the onset of a cytokine storm and programmed cell death. Thus, Zn^2+^ assists host immunity in outpacing viral multiplication and the commonly lethal inflammatory response.

## Mammalian cathepsin B illustrates pharmacotherapeutic intervention against host protease involvement in infectious and non-infectious inflammation by Zn^2+^ and Zn^2+^-interactive agents

The severity or lethality of COVID-19 depends upon the intensity of the inflammatory response. Zn^2+^-sensitive proteases listed in Table 1 have been implicated in the viral life cycle and/or the host response to infection. The “polypathogenic” background of excessive cathepsin B activity provides a straightforward illustrative model for the “polytherapeutic” interaction of metformin and Zn^2+^. A comprehensive review would require a book-length tome. Many viruses can hijack various host proteases. Cathepsins B and L have both been implicated in COVID-19 and other viral myocarditis (Wang et al. 2018; Hashimoto et al. 2021; Zhao et al. 2021; Ding et al. 2022; Huang et al. 2022); and both exhibit Zn^2+^ responsiveness ex vivo. Viral and host cysteine proteases sharing the same compartment also share the same exposure to exchangeable Zn^2+^.

The inflammatory system is difficult to distinguish from the endocrine system; and cathepsin B responds to both. Inflammation involves extracellular/intracellular, receptor-mediated signaling by cytokines and proteolytic execution. The full significance of cathepsin B in non-infectious inflammation has not yet been realized; recent information is startling (reviewed by Xie et al. 2023). In all human cells cathepsin B is present in large amounts. This primordial protease is no longer considered to be an exclusively lysosomal activity; its presence in the cell nucleus and extracellular space is puzzling. Low reaction rates are involved in housekeeping functions; higher reaction rates are involved in reversible or irreversible inflammation and programmed forms of cell death. Cell Zn^2+^ regulation is compartmentalized. Under fluctuations in adequate intracellular content/concentration, compartmental Zn^2+^ might be part of the buffer maintaining submaximal reaction rates of intra-lysosomal and extra-lysosomal protease activities. However, inadequate Zn^2+^ might weaken the buffer and allow uncontrolled hyper-catabolism involving cathepsin B.

Much of the cell content of diverse proteases exists as pro-proteases with an inactivating pro-region. Self-cleavage of the pro-region overlying the catalytic Cys-His partners can cause cathepsin B to auto-activate. Auto-activation responds to the same factors that increase the reaction rate of mature cathepsin B and other proteases. Pro-proteases can also be activated by the convertase action of surrounding ongoing protease activities. It remains to be determined whether cathepsin B is among trigger proteases. Regardless, hyper-catabolism of convertase activities can increase the active fraction of diverse proteases. Beyond some reaction rate, acceleration of pro-protease activation and positive feedback (or “feed forward”) is suspected. A disruptive effect of lowered Zn^2+^ on the vacuolar membrane system might be somehow related to positive feedback (item 4 above). Much background from gene knockouts implicates cathepsin B in non-lethal inflammatory pathogenesis and programed death. In various cell types, cathepsin B can mediate housekeeping functions at moderate reaction rates, reversible inflammation at higher rates and programmed death when unleashed (reviewed by Campden and Zhang 2019; Xie et al. 2023; Xu et al. 2023). Gene deletions reveal that cathepsin B hyper-catabolism and/or loss of compartmentalization contributes to the tissue pathogenesis of many infectious and non-infectious diseases. Thus, partial suppression of cathepsin B is necessary to maintain the life of the cell.

Knockout of the gene for an enzyme can be compared to complete “whole body” inhibition of that enzyme in all specialized cells. Although pro-cathepsin B zymogen is expressed in large amounts, deletion of its gene is phenotypically silent due to functional redundancy of other cathepsins. The cathepsin B gene provides a large store of inactive protease zymogen for mobilization in response to inflammatory stimuli such as TNFα. Cathepsin B gene knockout protects against lethality resulting from viral myocarditis and inflammasome activation (Wang et al. 2018, reviewed by Scarcella et al. 2022). All nucleated mammalian cells have receptors for TNFα. Cathepsin B gene deletion protects against tissue damage and death from injection of TNFα in uninfected mice (Guicciardi et al. 2001). Post- receptor signals linking TNFα to cathepsin B hyper-catabolism in various cell types are unknown; however, this protease is a major player in the execution of myocardial tissue injury from pro-inflammatory cytokines that are operative in COVID-19. TNFα is a major pro-inflammatory cytokine that is associated with non-infectious heart disease (Yuan 2020a, b). TNFα is also associated with COVID-19 outcomes (Adeghate et al. 2021, Zawawi et al. 2023). COVID-19 has been linked to a high incidence of inflammatory heart disease and heart failure, i.e. “post-COVID heart syndrome” (reviewed by Akhmerov and Marbán 2020; Adeghate et al. 2021; Gasecka et al. 2021; Sidik 2022; Xie et al. 2022; Sewanan et al 2023 and others).

The Zn^2+^ responsiveness of cathepsin B in ex vivo assay is consistent with the bio-assayed effect of Zn^2+^ in controlling intracellular myocardial proteome degradation (Fig. 4, reproduced from Lockwood 2010 and see below). Cathepsin B was preliminarily dialyzed against EDTA to remove metals and air-oxidized by reversible oxidation of its catalytic cysteine sulfhydryl to presumptive sulfenic acid (protease-SO). Several mM DTT reduces and reactivates cathepsin B. Millimolar experimental Zn^2+^ concentrations can aggregate or precipitate many proteins, thereby inhibiting enzymes artificially (Poulson et al. 2020). However, relevant biological Zn^2+^ concentrations can naturally influence the higher-order structure and/or reaction rates of many enzymes. The inhibitory EC-50 of Zn^2+^ for cathepsin B is approximately 1 µM under artificially maximized conditions of reaction rate (i.e. pH 5.5, DTT = 5 mM, following removal of metals by chelation). DTT binds Zn^2+^ and decreases its effective concentration. Under these conditions, a 100-fold span of minimally to maximally effective inhibitory Zn^2+^ concentrations is approximately 0.1 µM to 10 µM. Intracellular concentrations of exchangeable Zn^2+^ fluctuate around several µM. The exchangeable Zn^2+^ concentration in various compartments depends upon metabolites and macromolecules of the enormous Zn^2+^ interactome as well as regulation of Zn^2+^transport. Thus, small fluctuations in the interactive Zn^2+^ concentration can increase or decrease the reaction rates of cathepsin B and perhaps many other Zn^2+^-responsive proteases.

## Naked ligands differ from metallo-pharmaceuticals

Pharmacology textbooks have no chapter titled “Naked Metal Ligands”. Multiple meanings are attached to terms such as: metallophore, metal carrier, metal shuttle, metal chaperone, metal co-transporter, metallo-pharmaceutical, metalation, mis-metalation etc. There is no unanimous chemical or biological concept of endogenous zincophores or pharmacological counterparts. Metformin is a naked metal ligand that is referred to here as a metallophore or zincophore.

A metallo-pharmaceutical consists of stable metal-containing drug administered as a preformed structure that retains the metal during its action, e.g. *cis*-platinum (cis-diamminedichloroplatinum II). Metallo-pharmaceuticals can be produced with non-biological metals. If a metallo-pharmaceutical retains its metal, then it can cause its action without changing the concentration, chemical properties or function of endogenous metals. In contrast: some drugs can be administered as naked ligands that associate and dissociate with endogenous metal cations after administration by ligand exchange in the gut, and as they travel through and out of the body. Some naked ligands might change the natural functions of endogenous metals or create non-natural functions.

Xenobiotic metallophores have much in common with natural biomolecules that bind metals. The molar cell content of Zn^2+^-binding metabolites and macromolecules is greater than the molar content of biological Zn^2+^. A naked pharmacological metallophore adds another competing ligand to the endogenous interactome. Natural or xenobiotic carriers can acquire a metal cation upon administration and yield some or all of the metal upon excretion. Any effect on body metal content depends upon the balance between input vs. output. Renal and hepatobiliary routes can reclaim some of the metals from endogenous or xenobiotic substances. A dissociable zincophore that increases Zn^2+^ absorption more than it increases excretion might deliver additional Zn^2+^ to various compartments and many binding sites. The same can be stated for natural dietary biomolecules of the Zn^2+^ interactome. Conversely, a naked ligand that is not absorbed can deplete body metals; diverse natural substances and drugs can increase excretion.

From the ancient Greek, a metallophore is “that which carries the metal” (Scavo and Oliveri 2022). That concept is true but incomplete. Reversible and irreversible metal binding can do far more than carry the metal. Metallophores can change the biokinetics and biodynamics of natural metal interactions with living systems and create non-natural interactions. To some extent the majority of drugs interact with metal cations by various chemical forces. Indeed, a metal cation can interact electrostatically with the charge asymmetry of almost any dipole. The Human Metabolome Database for 2022 has increased the number of metabolites to 217,920 (Wishart et al. 2022). Many or most endogenous metabolites are somehow interactive with metal cations via various attractive vs. repulsive forces (Álvarez-Fernández et al 2014; Bellotti 2021).

Nature was a student of coordination chemistry and ligand exchange. Nicotianamine, staphylopine and yersinopine are hexadentate counterparts of EDTA in plants and bacteria (Seregin & Kozhevnikova 2023); others might be discovered. For practical purposes, a metallophore might be distinguished from other metal-interactive substances by the nature of the interacting forces, e.g. affinity, denticity, reversibility, and on-off kinetics of the dative bond; however, such differences are not well defined. The fraction of Zn^2+^ that is transported across various membranes as the independent cation vs. the fraction that is co-transported as bound to other endogenous substances is presently unknown. It seems likely that at least some Zn^2+^ absorption from the gut is mediated by co-transport with carriers from foodstuffs. A dissociable ligand such as metformin can acquire a metal cation beginning in the gut, undergo many exchanges during its trip through the body and then yield most or all of its metal back to the body under reclamation by renal and hepatobiliary pathways. Zn^2+^, citrate and other metabolites engage in peculiar interactions with uncertain significance (Sauer et al. 2020). Transport processes involved in both input and output of metals from all compartments are under homeostatic transcriptional control of many proteins involved.

As a broad operational definition for present purposes: *A metallophore is an agent that reversibly binds to a metal so as to possibly modify (a) its chemical properties and (b) its interactions with biomolecules and life processes.* The two parts of this definition are redundant because any agent that binds to a metal cation might also influence its chemical characteristics, biomolecular interactions and biokinetics. Figure 3 illustrates that the methyl groups of metformin impose partial hydrophobicity upon Zn^2+^ and other metal cations. They simultaneously decrease the solvent attraction for two ligand-binding sites and promote non-specific compatibility with hydrophobic sites. Various metallophores can increase or decrease interactions of metal cations with active and passive transport processes, signaling, transcription, enzyme activities, structures etc. The biological effects of pharmacological metallophores are potentially as numerous and diverse than the many natural roles of metals. In theory metal binding might decrease the concentration-dependent downhill passage of metal cations through ion channels. However, *if a metallophore is actively transported by an energy-dependent process, it can increase the co-transport of a bound metal against its concentration gradient.* Some metallophores can also serve as protonophores. The effects of metformin on body contents/concentrations of Zn^2+^ and other metals are unknown.

## Stable preformed metallo-pharmaceuticals are under investigations as anti-viral agents

Preformed metal coordination complexes are under investigation as anti-viral agents acting by various proposed mechanisms (e.g. Marzo and Messori 2020; Karges and Cohen 2021; Pal 2021; Ioannou and Vlasiou 2022; Mehrotra et al. 2023 and others). In various investigations, the distinctions between metallo-pharmaceuticals and naked ligands is often unclear. A variety of Zn^2+^-binding substances are being investigated against COVID-19 at various concentrations under various conditions, e.g. pyrithione, pyrrolidine dithiocarbamate, hinokitiol, quercetin, tropolone and thiotropolone and others (Lee et al 2009; Zhao et al 2017; DeLaney et al 2021; Di Petrillo 2022; Tao et al 2022). Proteasome inhibition with various pre-formed metal complexes is an active area of research (Ahmad 2020). The effects of these investigational substances on the exchangeable concentrations of Zn^2+^ and other metals in various cell types are unknown. Therefore, it is not known whether their effects are attributable to the metal complex or a change in independent metal concentration(s) or both; the same uncertainty applies to metformin. Pre-formed Zn^2+^-pyrithione can inhibit proteasomal function in cultured cells (Table 1). Interestingly, the pre-formed Zn^2+^-pyrithione complex has been shown to inhibit viral multiplication in cell culture and protease activities (Kladnick et al. 2022). It remains to be determined whether the Zn^2+^-pyrithione complex is a direct protease inhibitor or releases independently inhibitory Zn^2+^. The drug that “cured” acrodermatitis enteropathica by enhancing Zn^2+^ absorption (diodoquine, see above) is under consideration against COVID-19. Long-term administration of either pyrithione or diodoquine to humans causes toxicity that does not result from increased Zn^2+^ alone. In contrast, metformin has combined characteristics conferring efficacy and safety in the absence of pre-existing organ dysfunction.

## Metformin safely improves the outcomes of COVID-19 and an amazing variety of non-infectious diseases; how might this unique “polytherapeutic” actions of a naked Zn^2+^ ligand be explained?

Metformin is a unique drug with unexplained benefits against infectious and non-infectious diseases caused by different primary etiologies (Ala and Ala 2021; Kim et al. 2022a; Goto and Perencevich 2023; Raza et al. 2023 and many others). The undefined processes familiar as low-grade and hi-grade inflammation involve interactions between many cell types. Inflammation somehow contributes to diverse causes of diverse diseases involving all organ systems. At elevated reaction rates most of the Zn^2+^-responsive proteases of Table 1 are involved in reversible or lethal inflammation involving various cell types; however, signals elevating reaction rates are uncertain (Guicciardi et al. 2001, Wang et al. 2018, reviewed by Scarcella et al. 2022). Notwithstanding oversimplification, “whole body” moderation of the Zn^2+^-interactive protease web of diverse cells can somehow decrease the execution of inflammation in response to stimuli and signals. Whole body moderation of the two viral cysteine proteases can simultaneously inhibit viral multiplication and invasion and decrease the pathogenic host inflammatory response while immunity develops.

It is well known that Zn^2+^, Cd^2+^ and Hg^2+^ (group IIB elements) are inhibitors of cysteine proteases with greatly differing potencies. Standard assays involve EDTA pre-treatment of the protease or inclusion in the reaction in order to maximize activity. However, has not been recognized that the biological functions of diverse proteases are simultaneously moderated by fluctuations of exchangeable intracellular Zn^2+^ concentrations. Zn^2+^ transport and the Zn^2+^ interactome influence its interactive concentration in various subcellular compartments. Compartmental regulation of an adequate amount of cell Zn^2+^ maintains proteases functioning at submaximal reaction rates. Under Zn^2+^ adequacy, “normal” regulation can allow the sub-lethal reversible hyper-catabolism that accompanies non-lethal inflammation. However, severe Zn^2+^ deficit and/or dysregulation can unleash the lethal inflammation and programmed death illustrated by acrodermatitis enteropathica. The exact responsiveness of the proteases of Table 1 to fluctuating cell Zn^2+^ concentrations are unknown. Allosteric Zn^2+^-binding sites of several caspases might stop their reactions at minimal Zn^2+^ concentrations that do not suppress less sensitive proteases (Perry et al. 1997; Chai et al. 1999; Eron et al. 2018). Speculatively, reversible inflammatory proteolysis under moderate Zn^2+^ deficiency might be mediated by proteases that are less inhibited by Zn^2+^ than the caspases that initiate irreversible inflammation and programmed cell death under severe Zn^2+^ deficit.

Background suggests that metformin interaction with Zn^2+^ can slow the viral life cycle and the pathogenic inflammatory response, thereby allowing acquired immunity to clear the infection before a cytokine storm imposes programmed cell death. Metformin might synergize the natural moderating effect of Zn^2+^ on viral proteases and a web of host proteases by serving as (a) a dissociable Zn^2+^ carrier modifying the natural transport, regulation and cell content/concentration of Zn^2+^ or (b) a direct modifier of the chemical properties and interactions of Zn^2+^ with proteases with no change in Zn^2+^ concentration or (c) both.

## Coordination chemistry and ligand exchange suggest that the separate therapeutic benefits of metformin and Zn^2+^ are somehow related: back to basics

Before 1900 it was known that multiple ligands can interact with binding sites of a central metal cation. What has come to be known as coordination chemistry was begun by Werner around 1900, i.e. “before Schrodinger” (reviewed by Constable 2019). In the 1920s Lewis suggested that the ligand is an electron donor; and the metal cation is an acceptor forming a dative covalent bond with both electrons donated by the ligand. The Nobel prize in chemistry was awarded to Kohn and Pople 1998 for theoretical advances that led to modeling of chemical structure using “first principles”. Computational chemists can now model chemical structures without enduring a malodorous laboratory. The computed structure of the metformin–Zn^2+^ complex reveals molecular features that cannot be envisioned by the Lewis principle (Figs. 1–3). Conversely, experimental chemistry reveals properties that cannot be computed. A combination of empiricism, theory and intuition supports the present hypothesis.

Guanidine binds Zn^2+^ (Aoki et al. 2002, Stanek et al. 2017); biguanide (guanyl guanidine) has greater affinity for Zn^2+^. The neighboring lone electron pairs at the 2 and 4 imino nitrogen positions of biguanide comprise a bidentate coordinating site for all endogenous metal species and many non-biological metals (Rusanov et al. 2022). The stabilities and lifetimes of various metformin–metal complexes depend upon metal affinities and association–dissociation kinetics. Metal-binding biomolecules have various denticities and affinities. A bidentate complex between metformin and a metal cation can leave multiple binding sites unoccupied and interactive with solvent or additional ligands. Endogenous metal cations can undergo dynamic exchange among water, the multitude of endogenous ligands and metformin. Metformin can also form mixed complexes with a separate molecule coordinated around a central metal. Mixed metal complexes with metformin (i.e. heteroleptic complexes) can include many metabolites or macromolecules including proteases.

Figures 1–3 are models of the “in vacuo” structure of the 1:1 metformin–Zn^2+^ complex. Experimental evidence indicates that the imino nitrogens retain their protons as seen through the mesh. The charge of isolated Zn^2+^ is 2+; however, the coordinated cation acquires electron density transferred from the bidentate ligand. In Fig. 2 the surface potential is color-coded from negative to positive by red, orange, yellow, green, blue. The small blue surface region is the coordinated Zn^2+^ cation partially masked by charge transfer from metformin. This coordinated Zn^2+^ can become a central cation sandwiched between the imino nitrogens of biguanide and the Cys(thiolate)-His(imidazole) catalytic partners of all cysteine proteases (discussed in Lockwood 2019).

A second metformin can bind in a 2:1 complex. The stepwise formations of 1:1 and 2:1 metformin–metal complexes create a complicated equilibrium that is dependent upon pH and the concentrations/concentration ratios of all participants. The successive stability constants of the metformin–Zn^2+^ complex have not been characterized under a biological mixture or defined conditions. The intrinsic dissociation constants for [metformin–Zn]^2+^ and [(metformin)_2_-Zn]^2+^ are not known at any pH. The distribution among 1:1 or 2:1 metformin–Zn^2+^ complexes in vivo depends upon the relative and absolute concentrations of metal and metformin, pH and many endogenous ligands that compete with binding of a second metformin ligand. In theory, the binding of the second metformin to the same metal cation has a lesser formation constant than the first. Estimated therapeutic intracellular concentrations of metformin are 10–100 μM; most estimates are below 50 μM. Under therapeutic concentrations the 1:1 metformin–Zn^2+^ complex and mixed complexes with endogenous biomolecules probably exceed the 2:1 metformin–Zn^2+^ complex by far. Interestingly, some combination of the 1:1 and 2:1 Zn^2+^ complexes of pyrithione around a central Zn^2+^ inhibits several protease activities (Kladnick et al 2022 and Table 1) with dissociation constants in the range of 10^−6^ to 10^−5^ (Lofts 2009). It is not known whether this inhibitory action is attributable to the 1:1 or 2:1 pyrithione–Zn^2+^ complexes or the dissociated Zn^2+^ or both. Similar uncertainties apply to metformin–Zn^2+^ complexes. Micromolar concentrations of Zn^2+^–pyrithione have anti-viral actions (Krenn et al. 2009); however, its toxicity discourages human use.

Solvated or aquo Zn^2+^ has been modeled to have 6 hydrogen-bonded water molecules in the first solvation shell (Cauët et al. 2010); the present computation agrees (Fig. 3). The second shell has been modeled to have a variable number of 12-14 waters; and the third has several hundred. Beyond three shells Zn^2+^ has little influence on the structure of water (Cauët et al. 2010). The images of solvated Zn^2+^ in Fig. 3 represent the structure of the first hydration sphere as viewed from separate perspectives. Many aquo metal cations [M(H_2_O)_6_]^Z+^ have an octahedral structure.

Human tissues are not believed to appreciably metabolize metformin. Bacteria of the gut might slowly convert a small amount to guanylurea through intermediate structures (Tisler and Zwiener 2019). However, nearly the entire dose can be recovered unchanged. Guanylurea can coordinate Zn^2+^ and other metals (Gungor et al. 2020); although its pharmacology is not well studied. Despite the absence of appreciable metabolism, metformin can be reversibly transformed into many different structures during its transit through the body**.** Metformin–metal coordination can begin in the gut before absorption and proceed through many ligand exchanges under the non-equilibrium conditions of the mammalian body. Metformin–metal complexes are dissociable after absorption from the gut. Zn^2+^ can reversibly exchange among hydrogen bonded water, metformin and many endogenous ligands. However, it is not known whether all interactions of the metformin–metal complexes with biomolecules are reversible upon drug elimination.

## Principles of coordination chemistry and ligand exchange underlie the dynamic partition of Zn^2+^ between solvent, the endogenous Zn^2+^ interactome, and the metformin zincophore

Associative and dissociative ligand exchange and the reversible formation of mixed complexes determine metformin influences on the biokinetics and biodynamics of metals. The formalities of ligand exchange can be found in textbooks of inorganic chemistry, including chemical kinetics (not discussed here). Some points have been applied to biology by Costello et al (2011); however, many questions remain.

For unknown reasons, many of the papers cited here begin with the statement that the “free biological Zn^2+^ concentration is in the picomolar range”. The significance that authors attach to this statement is uncertain. However, it ignores the fundamentals of solvation and ligand exchange. “Non-free” Zn^2+^ need not imply “non-interactive” Zn^2+^. Truly free Zn^2+^ can exist only in a vacuum. Zn^2+^ in water is certainly not free; it has six hydrogen bonded waters of solvation [Zn(H_2_O)_6_]^2+^ (Fig. 3). It has long been debated whether hydrogen bonds between metal cations and water have partial covalent character (reviewed by Extance 2017); however, this need not be relevant here. The solvent is excluded in equations of chemical equilibrium in water; however, solvent is a bonafide participant in reactions. Hydrogen-bonded water can be interpreted as one more ligand of metal cations which undergoes continual exchange with other ligands.

Hexadentate chelators can occupy and block all six ligand-binding sites of a metal cation with six intra-molecular dative bonds. Most endogenous metal coordinating substances are not hexadentate. Bidentate coordination can leave 4 sites exposed or readily exchangeable and interactive. In general, hexadentate coordination has greater affinity than bidentate coordination due to the combined effects of multiple bonds between a single molecule and all ligand sites of the metal. In theory, a metal can be released from a metallophore by dissociation into solvent, or direct transfer to another metal-binding substance, or enzymatic cleavage of the metallophore and destruction of its multi-denticity, or conceivably a conformational change.

A Zn^2+^ cation with some of its 6 sites occupied by ligands other than water is correctly defined as “non-free”; however this does not imply non-interactive. A 1:1 bidentate ligand can leave 4 sites solvated or occupied by exchangeable other ligands. Indeed, Zn^2+^ that is defined as “non-free” can be more interactive with some ligands than Zn^2+^ that is defined as “free”. A more useful concept than the small cellular concentration of “free” Zn^2+^ is the concentration of ligand-binding sites that are exposed or readily exchangeable with solvent or other ligands. The cellular concentration of readily exchangeable ligand-binding sites of Zn^2+^ is at least several micromolar. This is approximately a million times higher than the picomolar concentration range of completely solvated or “free” biological Zn^2+^ that is commonly believed to have 6 waters of solvation. In theory, bound Zn^2+^ can be released into solvent before being accepted by other binding sites. However, metabolites, transporters, transcriptional regulators, enzymes and the vestibules of ion channels can accept Zn^2+^ by direct transfer from another binding site; i.e. the Zn^2+^ need not become “free” or solvated before transfer.

With associative ligand exchange the incoming and leaving ligands are interactive with separate binding sites of the metal at the same time. A transient unstable reaction intermediate forms between the metal and the incoming and leaving ligands. Associative ligand exchange permits a metal cation to be interactive with molecule B while it is simultaneously bound to molecule A by forming the unstable transient intermediate: [A–M^n+^–B]*. Therefore, a metal can be directly and reversibly transferred between B and A without first passing through a solvated intermediate with six waters. Thus, the low concentration of intracellular Zn^2+^ that many authors define as “free” need not be relevant to Zn^2+^ transfer between binding sites of a drug and an endogenous biomolecule.

In dissociative ligand exchange the transient intermediate with two simultaneously bound ligands is forbidden. The leaving ligand must first dissociate before the incoming ligand can independently interact with the metal cation, i.e. Zn^2+^ must first become transiently “free” or solvated. Transfer of Zn^2+^ between binding sites by dissociative ligand exchange depends upon the limiting on-off kinetics as well as affinities and concentrations of all participants. Thus, solvation need not be the rate-limiting step in transfer between ligands. Solvent water can exchange very rapidly. The kinetics of metal passage through the solvated state can be rapid; and need not be rate-limiting in transfer. The solvated intermediate need not cause appreciable delay of many biological transfers. However, with some ligands the “on-off” kinetics might be more biologically important than the final equilibrium state (Creutz et al 2006). It appears that metal exchange between metformin and all endogenous ligands of metals is fast in comparison with the transfer of this non-metabolized drug into and out of the body.

A third possibility is the formation of a stable mixed complex with multiple ligands coordinated around a central metal cation. The simultaneous binding of two or more ligands to separate binding sites of a central metal cation need not be transient or forbidden. The dimethyl moieties of metformin might increase the association of Zn^2+^ with proteins by introducing additional binding forces or hydrophobicity (Lockwood 2019). However, “stable” does not imply forever. Stable mixed complexes can have a wide range of lifetimes and exchange kinetics. Many mixed complexes can form with a pharmaceutical ligand and an endogenous ligand, including a metal sandwiched between a biguanide derivative and the catalytic partners of many proteases (illustrated and discussed in Sweeney et al. 2003, Lockwood 2019).

## How does the uncomplicated structure of 1,1-dimethyl biguanide assist Zn^2+^ to inhibit diverse proteases?

Coordination can change the chemical properties and reactivity of metals. Beyond the present scope, much literature describes biguanide-metal complexes as catalysts for non-biological reactions in chemical engineering (reviewed by Bankar and Kathuriaet 2022). Zn^2+^ is actively inserted into some metalloproteins by metallo-insertases. A GTP-dependent chaperone/insertase system has been characterized (Chen and O’Halloran 2022). It is not known whether metformin might catalyze permanent Zn^2+^ insertion into some binding sites by decreasing the energy barrier that must be overcome. However, Zn^2+^ inhibition of proteases under defined conditions ex vivo is a spontaneous process requiring no cofactors or the energy input of metallo-insertases (Fig. 4).

One possibility is that the two methyl groups of metformin merely introduce a degree of hydrophobic character to coordinated metal cations. Metformin might non-specifically decrease solvent attraction. Partition of Zn^2+^ between solvent and a binding site can shift if the solvent attraction for Zn^2+^ is decreased. Metformin decreases solvent affinity for a metal cation by blocking two ligand-binding sites and replacing solvent attraction with two hydrophobic methyl groups as illustrated by the computation of Fig. 3. Thiolate-imidazolium catalytic partners have an attraction for the ligand-binding sites remaining exposed, and concentration-dependent association. By decreasing solvent attraction metformin might non-specifically increase the fraction of time that inhibitory Zn^2+^ is interactive with the CysHis catalytic partners. In addition, the dimethyl moieties might form a hydrophobic bridge between metal cations and localized “greasy spots” formed by constellations of hydrophobic amino acids surrounding the catalytic partners. Some biguanide derivatives might create [protease–Zn^2+^–metformin] “sandwiches”. Derivative moieties of some biguanide–metal complexes can also guide Zn^2+^ to a particular protease by creating specific interactions with multiple binding sites surrounding the catalytic partners (illustrated and discussed in Lockwood 2019 and see appendix). The Zn^2+^ complexes of biguanides such as phenformin can have anti-proteolytic specificity and potency by close mimicry of known substrate motifs.

The secret of metformin’s unique therapeutic success might include some combination of many attributes in addition to metal partition between solvent and binding sites, e.g. (a) required affinity for Zn^2+^ relative to other endogenous metal species (b) required affinity for Zn^2+^ relative to endogenous ligands of the enormous competing Zn^2+^ interactome (c) required “on-off” kinetics of all ligand exchanges (d) required intestinal bioavailability for Zn^2+^ binding (e) required interactions of the Zn^2+^ coordination complex with all absorptive and excretory transport machinery of liver and kidney (f) required selectivity(s) and lifetime(s) of mixed metformin–metal complexes with multiple targets such as proteases (g) required stability of the drug against metabolism (h) acceptable human side effects, and others. Metformin is not the only metallophore. Multiple pharmacological Zn^2+^ carriers or chaperones have been discovered; and some might prove to be therapeutically useful (Leuci et al. 2020); although safety is a concern. Metformin is a lucky accident that might be difficult to improve upon by a priori drug design.

## A “polytherapeutic” paradox: metformin actions against infectious and non-infectious diseases of unrelated primary etiologies

The dictionary defines a paradox as: “a seemingly absurd or self-contradictory statement or proposition that when investigated or explained may prove to be well founded or true”. It seems ridiculous to suggest that a single non-metabolized molecule can improve the outcomes of infections with viruses, prokaryotes and eukaryotes and also improve a large variety of non-infectious diseases. Nonetheless, rational trials have confirmed that metformin does have these paradoxical benefits against an amazing variety of unrelated infectious and non-infectious disease etiologies (reviewed from various viewpoints by Ala and Ala 2021; Grytsai et al. 2021a, b; Kim et al. 2022a; Goto and Perencevich 2023; Raza et al. 2023). Metformin has been repeatedly referred to as a “miracle drug”. In the history of pharmacology, there has never been a molecule quite like it. Metformin is now in long-term trials as an “anti-aging” agent. No single underlying therapeutic mechanism(s) has been convincingly demonstrated despite thousands of papers; indeed there might be no single mechanism. Importantly, the actions of metformin against hyperglycemia are not the same as its actions against protein degradation. Metformin can improve the inflammatory sequelae of type 2 diabetes over the years without improving hyperglycemia. Accordingly, research on the anti-inflammatory/anti-viral actions of biguanides must be distinguished from the anti-diabetic actions.

A rational explanation might be found by comparing the polytherapeutic “magic” of metformin with Zn^2+^ biology and the undefined process known as inflammation. The relationships between total body Zn^2+^ content, plasma/serum Zn^2+^ measurements and exchangeable intracellular Zn^2+^ concentrations in various cell types are presently unknown; and the effects of metformin on these relationships are a mystery. However, it is known that hyper-catabolism of a network of proteases (Table 1) can cause or contribute to inflammation and many diseases caused by different primary etiologies. Hyper-catabolism can be caused by excessive expression of proteases and/or insufficient expression of proteinaceous inhibitors or excessive peptidolytic reaction rates. Disruption of membrane structure and subcellular compartmentation is a separate factor. Zn^2+^ deficiency contributes to many skin diseases in addition to acrodermatitis enteropathica (Raza et al 2023; Tiffany et al 2020; Zou 2023). Various diseases that can be improved by metformin appear to be worsened by Zn^2+^ deficit, including type-2 diabetes, many dermatological conditions, and heart diseases etc. (reviewed by Costa et al. 2023, Hara et al. 2023). Correction of hyper-catabolism can be achieved by the inhibitory interaction of metformin with its metal partner (Fig. 4). Metformin is now being studied in relation to treatment of cardiomyopathies and other conditions as well as type-2 diabetes (see below). However, no connections between metformin, Zn^2+^, and protease activities have been recognized.

Metformin and other biguanides were discovered in the 1940s in the search of hundreds of thousands of chemicals for anti-malarial actions without attention to metal coordination. Hundreds of biguanide derivatives have been synthesized since the pioneering studies of Curd et al. (1945, 1946). Proguanil is a biguanide that is now widely used against malaria despite side effects. Older concepts involving only one mechanism of proguanil action are now under revision toward multiple uncertain mechanisms (Skinner-Adams et al. 2019). Metformin has anti-malarial actions in animals and humans although it is not presently used for that purpose (Miyakoda et al. 2018; Oriaifo 2018; Vera et al. 2019). The anti-malarial actions of biguanide derivatives might be partly associated with Fe^3+^-interactive inhibition of proteases in the intra-erythrocytic stage of the plasmodial life cycle (Sweeney et al. 2003). Intra-erythrocytic plasmodial growth requires degradation of hemoglobin and release of large amounts of oxidized iron. The liver stages also require protein degradation in the absence of high iron**.**

Viral, bacterial and eukaryotic microbes have vastly different life cycles. The paradox of a single drug against many different infections might be due to the fact that morbidity and mortality are commonly caused by two variables of infectious diseases: the load of infectious agent, and the pathogenicity of acute and chronic host defenses in responses to the infections. It is difficult to distinguish therapeutic biguanide actions against microbes from therapeutic biguanide actions against the host inflammatory host response (Ishida 1965; Foretz et al 2023; Goto and Perencevich 2023). Both of these variables might be improved by moderation of Zn^2+^-responsive proteases. However, innate host immunity to infections has more mechanistic similarities than the life cycles of various invaders. Benefits of metformin against diverse microbial infections might be primarily attributable to a decrease in the excessive inflammatory response, i.e. host-directed therapy.

Many viruses encode proteases (Sharma and Gupta 2017). However, the life cycles of viruses that do not encode proteases can involve host proteases (e.g. Bestle et al 2020). Although influenza viruses do not encode proteases, infectivity is activated by several host proteases. Host proteases have been suggested as druggable targets against influenza (Krenn et al. 2009; Böttcher-Friebertshäuser et al 2013; Beestle et al 2020, Rahbar Saadat et al. 2021). Some host proteases employed by influenza viruses are sensitive to Zn^2+^ and Cu^2+^ (Podsiadlo et al. 2004). Various biguanide derivatives inhibit multiplication of some viruses in cultured cells (Weinberg. 1968; Fara et al. 1974). The actions of biguanides against influenza infections might be associated with metal-interactive inhibition of host proteases involved in  viral life cycles and the resulting host inflammatory response. Zn^2+^ carriers such as pyrithione can inhibit the multiplication of corona viruses and other viruses in cell culture (Te Velthuis et al 2010). However, assay of viral multiplication in cell culture omits host involvement and does not provide an indication of the overall disease state.

Old imperfect studies indicate that some biguanides have preventive and therapeutic action against influenza in humans. Influenza viruses hijack host proteases. In 1950 it was reported that metformin (referred to as flumamine) improved outcomes in an influenza epidemic (Ey 1950). Metformin has since been found to have actions against a wide range of microbial disease states including tuberculosis (reviewed by Goto and Perencevich 2023). Moroxydine is a biguanide derivative that was introduced against influenza in the 1950s; however, it is now limited to veterinary uses (Sheppard 1994). A preventive effect of phenformin was reported in a 1970 flu outbreak (Berglund et al. 1970). It has recently been proposed that phenformin is a broad spectrum anti-viral agent (Renz et al. 2022). Inhalation of biguanide derivatives has been recently suggested in order to localize exposure to the lung and minimize side effects elsewhere (Lehrer 2020). Phenformin, moroxydine and proguanil have structural correspondences favoring interactions with the active sites of some proteases (discussed in Lockwood 2019).

Chlorhexidine is an anti-bacterial biguanide disinfectant that causes Zn^2+^-interactive protease inhibition (Cronan et al. 2006); it is used as a topical antiseptic mouthwash against periodontal disease. Chlorhexidine and alexidine are large molecules that are not absorbed; however, exposure to metformin through the bloodstream also decreases periodontal inflammation (Tseng 2022).

## Metformin coordination might modify the systemic, cellular and subcellular co-transport and exchangeable concentration of inhibitory Zn^2+^ by multiple mechanisms

Metformin adds a competing substance to the natural Zn^2+^ interactome beginning in the gut. Dissociable Zn^2+^- binding substances could change its absorptive input, distribution, exchangeable compartmental concentrations and output by renal and hepatobiliary pathways. This differs from direct protease inhibition by the metformin–metal complex, although both are probably operative. Zn^2+^ is co-transported with biomolecules ranging from familiar metabolites such as citrate to unusual hexadentate botanical and bacterial chelators that block all interactions (Haydon and Cobbett 2007; Kocyła et al. 2021, Stanton et al. 2022). A typical metformin dosage of 1–2 g/day might create artificial pathways of intestinal Zn^2+^ absorption, inter-organ transport, and distribution among systemic, cellular and subcellular compartments. Metformin might also compete with non-absorbable foodstuffs that bind intestinal Zn^2+^ and decrease bioavailability. Increased Zn^2+^ bioavailability combined with active transport of the metformin–Zn^2+^ complex might have combined effects on Zn^2+^ absorption. Following active uptake, dissociation of the metformin–metal complex in various tissues could release Zn^2+^ in various tissues.

Interestingly, plants actively transport metformin, and presumably its bound metals, from root to shoot (Eggen 2012; Nespor et al. 2020). Metformin might be a pharmacological counterpart of unknown endogenous Zn^2+^ carriers. In mammals, metformin is absorbed from the gut and transported between tissues by many active transporters. Human organic cation transporters (OCTs, SLC22) and multidrug and toxicant exporters (MATEs, SLC47) have been surveyed by (Koepsell 2020), including proguanil transport. Transport of metformin by these transporters has been reviewed by Sundelin et al. 2020, Li et al. 2022a, Shirasaka et al. 2022, Damanhouri et al. 2023. Genetic variability of OCTs and MATEs has been shown to be associated with variability in therapeutic response to metformin. In some species these transporters are known to move various metal cations. Human co-transport of metformin and bound endogenous metals by OCTs and MATEs has not been well studied; however, they do transport cis-platinum in the kidney. In addition, separate ATP-driven transporters can also co-transport metals bound to various substances. Due to binding to intestinal contents 50–80% of dietary Zn^2+^ is not absorbed (Maares and Haase 2020, Zake et al. 2021, 2022).

Studies using positron emission tomography qualitatively illustrate the pharmacokinetics of radio-labelled Zn^2+^ (Firth 2022) and radio-labelled metformin (Gormsen et al. 2016; Sundelin et al. 2020). Neither injected Zn^2+^ nor injected metformin are uniformly distributed among tissues as a function of time. These beginning studies imply that metformin and Zn^2+^ are actively accumulated and transferred between some of the same tissues. Renal and hepatobiliary excretory pathways apparently have sufficient affinities and capacities to reclaim most bound Zn^2+^ from the multitude of excreted substances. A complex system of metal homeostasis compensates for insufficiencies or excesses in all organisms. Transcriptional changes might adjust the status of absorptive and excretory transporters so as to partially offset pharmacological perturbations of Zn^2+^ input vs. output and restore balance.

Changes in the micromolar concentration range of exchangeable cell Zn^2+^ are difficult to accurately determine. Understanding of metformin action is limited by present understanding of inter-organ Zn^2+^ biokinetics and biodynamics. Measurements of serum or plasma Zn^2+^ within an individual can fluctuate by 22% on a daily basis (Hambidge et al. 1989); however, plasma proteins do not simultaneously fluctuate. Measured changes in total serum/plasma Zn^2+^ might be accompanied by much larger changes in non-sequestered exchangeable Zn^2+^ because only 2–8% of serum/plasma Zn^2+^ is non-bound as measured by ultra-filtration (Prasad and Oberleas 1970). Therefore, it seems that the intracellular/extracellular distributions of non-bound Zn^2+^ varies far more than 22% on a diurnal basis. In the same individual the range of variability of individual spot measurements of Zn^2+^ is very large. Moreover, intra-vascular Zn^2+^ is only 1–2% of total body Zn^2+^; therefore, spot measurements of plasma or serum Zn^2+^can be a misleading indicator of total body Zn^2+^. Epidemiological studies seeking a relationship between serum Zn^2+^ levels and various human conditions typically result in a regression line through a very large scatter of measurements. Such large statistical variability can be associated with multifactorial disease etiologies as well as the variability of spot determination of Zn^2+^. Exchangeable intracellular Zn^2+^ is merely 1-2% of protein-bound cell Zn^2+^. Exchangeable cell Zn^2+^ pools can change rapidly in response to extracellular Zn^2+^. Better biometric indicators of total and compartmental Zn^2+^ adequacy are needed.

Protons and metal cations compete for binding to biguanide. Formation of a biguanide–metal complex is pH-dependent; therefore, absorption from the gut might differ in the alkaline and acid segments of the GI tract. Metformin might acquire metal cations at cytoplasmic pH and dissociate them at intra-lysosomal pH due to greater proton competition. Speculatively, this property could create a transmembrane Zn^2+^- shuttle, increasing intra-lysosomal Zn^2+^ with little change in cytoplasmic metal concentration or total body metal content. An anti-lysosomal mechanism of various drugs against COVID-19 has been suggested, i.e. lysosomotropism (Blaess et al 2022).

## Non-responsiveness of many individuals to various actions of metformin is unexplained

Approximately 40% of individuals do not exhibit meaningful anti-diabetic or other benefits of metformin. Likely causes of unresponsiveness might involve Zn^2+^ deficit resulting from genetic variability in its regulation or dietary insufficiency or some combination. In addition, a high cell content of proteases or low content of proteinaceous protease inhibitors (Ćwilichowska et al. 2022) might cause severe pathogenic inflammation. Under Zn^2+^ deficit metformin might not have sufficient Zn^2+^-interactive, anti-proteolytic potency to overcome the hyper-catabolism of protease over-expression or inhibitor under-expression. A recent report indicates that oral Zn^2+^ administration increases the anti-diabetic benefits of metformin relative to metformin alone. Dosage of 1–2 g/day metformin with 25 mg Zn^2+^/day (50 mg every other day) increased total serum Zn^2+^ content above the level resulting from the same metformin dosage alone (Younis et al. 2022 and reviewed therein). In another study, metformin alone caused a slight increase in serum Zn^2+^ as compared to newly diagnosed type-2 diabetic subjects; however Cu^2+^ differed (Mohammad et al. 2021). These topics require more extensive investigation. Metformin effects on cobalt and iron are conflicting and not discussed here.

Recommended daily dosage of metformin is 1 to almost 2.5 g/day depending upon individual tolerance of gastrointestinal side effects. Absorption of dietary Zn^2+^ from the gut is 16–50% (Maares & Haase 2020); and absorption of a dose of metformin is approximately 60% (Zake et al. 2021, 2022). Much dietary Zn^2+^ is wasted due to the low bioavailability associated with binding to intestinal contents. The 60% fraction of metformin absorption decreases as the dose increases (Liang and Giacomini 2017). Speculatively, large doses of metformin might carry some fraction of bound intestinal Zn^2+^ out of the body, resulting in a biphasic dose dependency if dietary Zn^2+^ is limited. If dietary Zn^2+^ is insufficient, high doses of metformin might actually lower the body Zn^2+^ content by decreasing absorption and/or increasing renal and/or biliary excretion of bound Zn^2+^. This dose-dependent problem might be solved by simultaneous Zn^2+^ supplementation (see Younis et al. 2022). A recent study indicates that prolonged metformin decreases the incidence of iron deficiency anemia (Wu et al. 2023); however, the opposite has been stated. A range of metformin doses might differentially influence cationic iron and cobalt involved in hematopoiesis.

## Over-expression of web proteases might require a potent direct inhibitor of peptidolytic reaction rates to overcome hyper-catabolism

Although insufficient Zn^2+^ alone can be inflammatory, inflammation and programmed cell death can occur without Zn^2+^ deficit. Signaling of inflammatory pathogenesis and programmed cell death must be distinguished from their execution by the proteases of Table 1. Most tissues can greatly increase transcription of protease genes under various conditions. For example, lysosomal biogenesis coordinated by transcription factor EB (TFEB) can cause large induction of cathepsin activities and inflammation (Settembre et al 2011 and subsequent papers from this group). Transcription causing severe protease/anti-protease imbalance might cause tissue damage under regulation of the maximal possible amount of exchangeable Zn^2+^. Oral Zn^2+^ supplementation alone might be insufficient to compensate for over-expressed proteases or under-expressed inhibitors. Various biguanide derivatives have differing anti-proteolytic potencies. The two methyl groups of metformin do not confer maximal anti-proteolytic potency as compared to other biguanide derivatives, such as phenformin (see Lockwood 2019 and appendix). Unlike metformin, the inhibitory action of phenformin in bioassay does not require extracellular Zn^2+^ because intracellular Zn^2+^ is sufficient to fully activate its anti-proteolytic action. Structures such as the phenformin–Zn^2+^ complex are more complimentary to some protease active sites than metformin resulting in greater inhibitory potency (Lockwood 2019). Future biguanide derivatives might provide more effective protease inhibition for severe hyper-catabolism by forming more stable Zn^2+^ alignment between the catalytic partners and the drug (see Appendix).

### **Despite many uncertainties, bulk proteome degradation responds to the endogenous range of extracellular Zn**^**2+**^**concentrations in a reliable bioassay: new significance of old findings**

Long before COVID-19, our lab accidentally became aware of a relationship between Zn^2+^ and major pathways of cell protein degradation. Our initial interest grew from attempts to improve the experimental longevity of the isolated perfused rat heart for use as a bioassay of protein degradation. In recent years our findings have become relevant to the etiology and therapy of various forms of cardiomyopathy. It is now known that Zn^2+^ deficiency causes or contributes to various human heart diseases (citations below).

Metabolic and osmotic artifact of cultured cells can cause large fluctuations in the rate of degradation. Therefore, we developed a unique bioassay for reliable minute-to-minute measurement of bulk degradation using non-recirculating perfusion of the ^3^H leucine-labelled rat heart (methods described in Lockwood 1996, 2010; Sweeney et al. 2003). Unlike cultured cells, the functional myocardium immediately signals the onset of injury or experimental artifact by contractile dysfunction, i.e. changes in heart rate, rhythm, intra-ventricular pressure development (dP/dT) or peak intra-ventricular pressure. The release of ^3^H leucine from biosynthetically labelled proteins was determined on a minute-to-minute basis in non-recirculating “one-pass” perfusate collected in fraction collector tubes. Contractile function was simultaneously monitored with a catheter in the right ventricle. Constant concentrations of experimental agents were infused into the flowing perfusate in a small volume of 200-fold concentrated solutions from infusion pumps. At the non-recirculating trans-organ flow rate that we employed, intracellular and extracellular ^3^H leucine pools exchange within 30 s. Under constant delivery of non-radioactive leucine at physiological concentration, a negligible amount of radiolabeled postcursor is reincorporated. This was determined by similarity of release before and after stoppage of protein synthesis with cycloheximide. Under this experimental protocol, the 100% control rate of ^3^H leucine release degradation corresponds to a bulk basal degradative rate of approximately 3%/h.

Krebs and Henseleit pioneered the classical myocardial perfusion buffer that bears their name. In contrast to other ions, they found that contractile function was the same with and without extracellular Zn^2+^ over limited periods. Their results indicated that the Zn^2+^ concentration difference across the plasma membrane has no immediate effect on the membrane potential or beat-to-beat function of the contraction/relaxation cycle. This contrasts with extracellular concentrations of other ions. Until this millennium it was believed that intracellular-extracellular Zn^2+^ exchange occurs only by binding to membrane proteins and/or fluid endo-exocytosis. Prior to current knowledge of Zn^2+^ transport, we confirmed that basal contractile function of the perfused rat heart is indistinguishable with and without extracellular Zn^2+^ in the perfusate over several hours; the electrophysiology of myocardial Zn^2+^ remains unexplained. Basal protein degradation was also the same with and without physiologically equivalent concentrations of perfusate Zn^2+^. In laborious background development we determined that ex vivo functional longevity of the perfused heart was greatly increased by endogenous concentrations of complete amino acids, citrate, pyruvate, lactate, glucose and the interstitial (extra-vascular) albumin concentration (Lockwood 2010). (Extravascular albumin concentration is only 5% of plasma albumin concentration). We suspected that the addition of Zn^2+^ to the perfusate might improve contractile function during extended periods of perfusion. Much to our surprise, we unexpectedly found that proteome degradation is quite sensitive to slight elevation of extracellular Zn^2+^ over extended periods of exposure. Following an initial delay, proteome degradation was slowly decreased by an extracellular Zn^2+^ concentration that had no effect on contractile function or ex vivo experimental longevity. Indeed, basal degradation appeared to be gradually inhibited by Zn^2+^ concentrations that were well within the endogenous range of Zn^2+^ fluctuations. Supraphysiological perfusate Zn^2+^ concentrations caused much greater decrease in degradation without initial delay.

Our interest in extracellular Zn^2+^ changed from technical development of perfusate composition to the basic control of proteome degradation by this cation. Plasma Zn^2+^ fluctuates by 20% on a diurnal basis (Hambidge et al. 1989). It is now known that intracellular Zn^2+^ exists in gradual slow communication with extracellular Zn^2+^ as regulated by membrane transporters operating in opposite directions. However, the electrophysiology of Zn^2+^ in relation to the relative rates of import vs. export, and the effect on contraction-relaxation remain puzzling. In short, we found that bulk proteome degradation is far more sensitive to fluctuations in the extracellular Zn^2+^ concentration than the contraction-relaxation cycle; this conclusion is valid despite all possible sources of artifact.

We devoted much effort to simulation of the endogenous upper limit of exchangeable extracellular Zn^2+^ concentrations under our defined cell-free perfusate composition. We measured gross intracellular Zn^2+^ content per mg tissue dry weight by atomic absorption. The intracellular Zn^2+^ content of this preparation can be increased by elevation of the extracellular Zn^2+^ concentration. Elevated extracellular Zn^2+^ results in a rate of import that exceeds the maximal rate of export from cardiac myocytes. The physiologically equivalent extracellular Zn^2+^ concentration was approximated as the concentration that caused no detectable change in monitored contractile function, or gross tissue Zn^2+^ content, or proteome degradation over several hours of exposure (for methods, see Lockwood 2010); this implies equality of balanced Zn^2+^ import and export. Zn^2+^ loading of cardiac myocytes is a slow process that is proportional to the elevated extracellular concentration and duration of exposure above a threshold and after an initial delay i.e. concentration-time integral exposure above threshold.

Eventual dysrhythmia caused by supra-physiological Zn^2+^ loading was reversible after a 20–30 min elimination period. In whole human plasma the Zn^2+^ concentration range is approximately 10–20 μM. Our use of the extra-vascular albumin concentration decreases a major Zn^2+^ binding intravascular substance by 95% (Bal et al. 2013). In our perfusate a Zn^2+^ concentration of less than 10 μM was determined to be supra-physiological. At 1 h, 3–5 μM extracellular Zn^2+^ caused only marginal inhibition of degradation. However, prolonged exposure to 5–10 μM Zn^2+^ began a slow gradual degradative inhibition after an initial lag period of approximately 10–20 min (shown in Lockwood 2010). Bulk proteome degradation could be inhibited by 20–30% with no detectable change in gross tissue Zn^2+^ content. Therefore it is the exchangeable cell Zn^2+^ pools that cause the inhibition. This responsiveness of proteome degradation to the endogenous range of Zn^2+^ fluctuations was not accompanied by any simultaneous changes in observable contractile parameters.

Bulk proteome degradation is partitioned between Zn^2+^ -inhibitable and Zn^2+^ -uninhabitable. The maximal inhibition of protein degradation caused by supra-physiological tissue Zn^2+^ loading was sustained at 75%. In contrast, 25% of bulk proteome degradation was completely insensitive to Zn^2+^ loading over extended periods. It seems likely that supra-physiological cell Zn^2+^ can stop most or all or the protease activities of Table 1. These proteases mediate much of lysosomal and extra-lysosomal degradation including proteasomal degradation. Complete inhibition of these proteases would eventually be lethal; and 75% inhibition cannot be sustained in vivo. Carboxyl protease and metallo-protease reaction mechanisms are not inhibited by Zn^2+^. The 25% of myocardial protein degradation that is resistant to supra-physiological Zn^2+^ loading is presumably mediated by these peptidolytic reaction mechanisms.

Zn^2+^ electrophysiology, and the role of Zn^2+^ in the contraction-relaxation cycle are not presently understood. Regulation of the myocardial Zn^2+^ concentration gradient across the plasma membrane by import and export are unexplained. Because the contraction-relaxation cycle is almost the same with and without physiologically equivalent extracellular Zn^2+^ it seems that the plasma membrane is slowly permeable to Zn^2+^, and this ion plays no major role in determination of the plasma membrane potential. Supra-physiological Zn^2+^ concentration eventually caused dysrhythmia; however, bulk protein degradation was inhibited at Zn^2+^ concentrations that caused no functional change. Although the exchange of intracellular and extracellular Zn^2+^ is not directly linked to the contraction-relaxation cycle, internal Zn^2+^ and Ca^2+^ are reportedly interactive by competition for an ion channel (Dorward et al. 2023). Increased extracellular Zn^2+^ can be taken up by the heart beginning in minutes as revealed qualitatively by positron emission photometry (Firth et al. 2022). Curiously, however, depletion of functional Zn^2+^ from the perfused heart in the absence of extracellular Zn^2+^ is a slow process. In the absence of extracellular Zn^2+^ non-sequestered intracellular Zn^2+^ pools are presumably maintained by a combination of defensive transport and release of Zn^2+^ from protein binding sites and degraded proteins. In any case, observable functional decline and elevation of proteome degradation in the absence of extracellular Zn^2+^ requires more than several hours.

In summary of this section, myocardial proteome degradation is inhibited by the endogenous range of extracellular Zn^2+^ fluctuations that caused no detectable change in contractile function. A role of extracellular Zn^2+^ concentrations in control of other cell processes has been suggested, e.g. inter-organ pancreatic -hepatic interactions (O’Halloran et al. 2013; Tamaki et al. 2013). Fluctuation in extracellular Zn^2+^ concentration might serve as an inter-organ “endocrine cation” interacting with hormonal factors that are known to control protein degradation.

## The gradual inhibition of myocardial proteome degradation by slight elevation of extracellular Zn^2+^ is accelerated and synergized by metformin

Metformin synergized the anti-proteolytic effect of extracellular Zn^2+^ exposure at relevant concentrations of both (Fig. 4). Biguanides can acquire endogenous tissue Zn^2+^. In the absence of added extracellular Zn^2+^ the therapeutic metformin concentration (25 µM) caused approximately 15–20% of total tissue degradation (Fig. 4). In the absence of metformin a sub-physiological Zn^2+^ concentration of 1 µM caused no change in basal degradation. However, initiation of sub-physiological 1 µM extracellular Zn^2+^ after metformin promptly synergized the inhibitory effect of metformin to 45–50% at the time illustrated in Fig. 4. Under longer time periods this synergistic inhibition progressed to approximately 60%. Such degradative inhibition was not accompanied by any detectable change in contractile function. This suggests that the concentration of exchangeable intracellular Zn^2+^ is slightly insufficient to fully activate the inhibitory action of metformin. Any possible increase in exchangeable tissue Zn^2+^ content was too small to be measurable against total bound Zn^2+^ (discussed in Lockwood 2019). Phenformin was approximately 100-fold more potent than metformin. Phenformin required no extracellular Zn^2+^ to inhibit lysosomal degradation because basal intracellular Zn^2+^ was sufficient to activate it. Phenformin was not cardiotoxic at and above the concentrations that inhibited lysosomal function.

The heart is now of particular interest in relation to Zn^2+^ deficiency. Both metformin and Zn^2+^ can improve human cardiomyopathy; however, no connection has been proposed. Firstly, Zn^2+^ deficiency alone can cause inflammatory cardiomyopathy (Rosenblum et al. 2020; Ozyildirim and Baltaci 2023). Secondly, the heart is a victim of COVID-19; and host cathepsins B and L are involved in the inflammation. Slight Zn^2+^ deficiency can change the proteome composition of the rat heart (Sun et al. 2023). Many transcriptional regulators have Zn^2+^-binding sites; however, the degradative component of turnover also plays a role in determination of proteome mass, composition and functional integrity.

## Whereas cell Zn^2+^ loading by elevated extracellular Zn^2+^ inhibits basal proteome degradation, a therapeutic Zn^2+^ blocking/excorporating agent greatly elevates it

In the absence of extracellular Zn^2+^ the time required for intracellular Zn^2+^ depletion exceeds the feasible experimental time. Therefore, antagonism of Zn^2+^ interactions with biomolecules was experimentally probed with a therapeutic dithiol chelating agent: 2,3-dimercapto-1-propanesulfonic acid (DMPS). DMPS is a permeant metal coordinating agent used in treatment of human Hg^2+^ poisoning (Djordjevic and Aaseth 2019; Blaurock-Busch 2020). DMPS is known to immediately block intracellular interactions of Zn^2+^ and Hg^2+^ with biomolecules at the therapeutic concentrations. The antidotal mechanism of DMPS involves immediate antagonism of Hg^2+^ or Zn^2+^ binding to biomolecules at high initial doses over several days; followed by gradual depletion of these metals over long-term administration of lower doses. DMPS coordination of both Hg^2+^ and Zn^2+^ immediately prevents their binding to biomolecules and then gradually promotes their export from cells. Our confirmed results with DMPS were not reported because DMPS has some affinity for other endogenous metals and does not specifically identify Zn^2+^. Nonetheless, DMPS has preferential affinity for Zn^2+^ in comparison with other endogenous metals. Human Zn^2+^ poisoning is rare; however, DMPS is an antidote against lethal Zn^2+^ toxicity in experimental animals (Llobet et al. 1988). Prolonged DMPS administration to humans for Hg^2+^ poisoning is known to cause Zn^2+^ depletion.

Infusion of supra-therapeutic, DMPS concentration caused a large, sustained elevation of the basal rate of protein degradation beginning within 1–2 min. ^3^H-Leucine release increased by approximately 50% within 20–30 min; and the elevation was sustained (confirmed unpublished observations). Lower DMPS concentrations cause lesser steady elevation after a delay. If DMPS was discontinued after an hour this 50% elevation returned to 100% baseline in approximately 30 min in the presence of 1 μM extracellular Zn^2+^ to provide repletion. Beyond 1 h the elevation gradually became irreversible in association with presumptive activation of pro-protease zymogens and disruption of membrane integrity. Over an hour, therapeutic DMPS concentrations caused reversible degradative elevation without injury at this time. Interactions of metformin and DMPS would be difficult to interpret and were not investigated. Supra-therapeutic DMPS exposure caused lethal structural damage to the heart in proportion to the concentration-time integral exposure.

Acquired Zn^2+^ deficiency or genetic dysregulation might cause or contribute to various heart diseases (reviewed by Rosenblum et al. 2020). A side effect of repeated DMPS is reversible skin inflammation that occurs in 2–20% of individuals. Thus, Zn^2+^ deficiency alone and therapeutic DMPS alone are both inflammatory. Skin inflammation is not caused by deficiency of other endogenous metals. Importantly, epidemiology now indicates that Zn^2+^ deficiency is associated with cardiomyopathies in the absence of infections (Braun et al. 2018; Yoshihisa et al. 2018; Yu 2018; Rosenblum et al. 2020; Hara et al. 2023; Meng et al. 2022; Kwon et al 2023; Ozyildirim and Baltaci 2023 and others). COVID-19 can cause inflammatory cardiomyopathy (Gasecka et al. 2021, Sidik 2022, Sewanan et al. 2023). Metformin has recently been found to have protective effects against various forms of non-infectious and infectious cardiomyopathies (Benes et al 2022, Kamel et al 2022, Li et al 2022b, 2023b and others). Integration of evidence from the in situ human heart and the perfused rat heart supports the anti-proteolytic hypothesis of metformin–Zn^2+^ interactions against inflammatory tissue damage.

## Results with the perfused heart do not support the safe use of chloroquine against COVID-19

Beyond the present scope, treatment of COVID-19 with hydroxychloroquine (Plaquenil®) and other lysosomotropic agents has raised much controversy (reviewed by Gasmi et al 2021; He 2022). Some investigators have suggested that chloroquine/hydroxychloroquine bind Zn^2+^; however, others strongly disagree (Kavanagh et al. 2022). Chloroquine does have anti-viral actions in cell culture at high concentrations (Vincent et al. 2005); however, multiple human trials indicate that safe hydroxychloroquine concentrations are ineffective against COVID-19 (meta-analysis of investigations by Di Stefano 2022). In routine usage for arthritis, slight chloroquine/hydroxychloroquine overdosage can cause chronic cardiomyopathy accompanied by undegraded protein debris in the extra-vacuolar space (Tönnesmann et al. 2013). However, the acute cardiotoxicity of chloroquine differs from the pathogenic effects of slight chronic overdosage. Chloroquine can cause immediate cardiac dysrhythmia and lethality. Chloroquine can be acutely cardiotoxic in treatment of COVID-19 (Gagnon et al. 2022).

High chloroquine or hydroxychloroquine concentrations are commonly used as experimental tools to selectively inhibit lysosomal function in cultured cells at concentrations that are lethal to mammals. In cultured cells, chloroquine disrupts the regulation of lysosomal osmolality, pH and volume control by unknown mechanisms, eventually causing massive cytoplasmic vacuolation (i.e. “foamy cytoplasm”); metformin does not cause this. Chloroquine concentrations required to inhibit 95% of lysosomal protein degradation are nearly 100-fold above safe therapeutic concentrations of less than 1 μM. We used high chloroquine concentration of 30 µM to experimentally inhibit lysosomal function, and not to characterize therapeutic mechanisms (Sweeney et al. 2003). The maximal anti-proteolytic action of chloroquine does not require Zn^2+^ or metformin. The current consensus is that effective anti-viral chloroquine concentrations can be hazardous; our experimental results with the perfused rat heart are consistent with this. In the perfused heart, the chloroquine concentration required to inhibit lysosomal function caused severe dysrhythmia that would not permit adequate function of the heart in situ. In contrast, the anti-proteolytic actions of metformin–Zn^2+^ clearly differ from chloroquine. Chloroquine inhibits only the lysosomal pathway even at very high concentrations. In contrast to chloroquine, the metformin–Zn^2+^ interaction can meaningfully inhibit some of lysosomal and extra-lysosomal pathways of degradation with no impairment of contractile function at therapeutically relevant concentrations.

## Can the independent benefits of metformin and Zn^2+^ against COVID-19 and non-infectious inflammation be additive if these agents are combined?

Metformin and oral Zn^2+^ supplementation separately improve COVID-19 outcomes by 42% and 40% respectively (Ben Abdallah et al. 2023; Bramonte et al 2023; Erickson 2023; Boulware 2023). The Zn^2+^-independent inhibitor of the viral main protease (Plavix®) improves outcomes by 80% when administered within several days of infection; however, later administration is not as effective (Reis et al. 2022, Lewnard et al. 2023). Thus, there is opportunity for improvement in all three. Clinical trials of outcomes can ignore basic mechanisms discussed above. However, therapeutic non-responsiveness to metformin or Zn^2+^ might be associated with insufficient action against viral and host proteases. Metformin is routinely administered at maximally tolerated dosage; and therapeutic concentrations are difficult to increase due to gastrointestinal distress. In contrast, the dietary intake of Zn^2+^ and resulting body content can vary greatly among individuals and can be increased over limited periods without harm. Firstly, it is conceivable that elevated Zn^2+^ intake above 50 mg/day over limited periods of administration might safely increase its demonstrated benefits against COVID-19. Secondly, it is likely that combined administration of metformin and Zn^2+^ might safely improve outcomes against the hazards of COVID-19 and perhaps some non-infectious conditions (Younis et al. 2022); safe dosage and dosage duration will have to be determined. Elevation of body Zn^2+^ content/concentrations alone by oral supplementation might supplement the natural effect of independent Zn^2+^ on the protease web. However, combined administration of metformin and Zn^2+^ might also create a non-natural protease inhibitor by formation of the metformin coordination complex (Figs. 1-3). It also seems possible that the independent anti-proteolytic effects of metformin and Zn^2+^ might be safely synergistic with the anti-proteolytic effects of Paxlovid®.

The optimal daily Zn^2+^ intake and resulting body content for prompt and effective therapeutic interaction with metformin might vary among individuals. Recommended dietary Zn^2+^ intake is 11 mg/day. However the Zn^2+^ contents of diets are variable, and dietary substances can decrease absorption (reviewed by Maares and Haas 2020). Intestinal bioavailability of Zn^2+^, metformin and the metformin–Zn^2+^ complex are uncertain under various diets. The fraction of intestinal Zn^2+^ that is bound to metformin and co-transported with it depends upon many competing metal ligands in foodstuffs (reviewed in Hall 2023). Incompletely absorbed Zn^2+^-binding drugs and common diuretics can also cause medically significant Zn^2+^ deficiency, e.g. the angiotensin receptor blocking drugs and thiazide diuretics (Macdonald et al 2012). Valsartan has a zinc-binding thiazole ring, and absorption from the gut is less than one third. Valsartan and hydrochlorthiazide are commonly combined against hypertension. Importantly, the percent of metformin absorbed decreases at high doses (see above and Liang and Giacomini 2017). As discussed above, the highest doses of metformin might carry some amount of bound Zn^2+^ out of the body, resulting in a biphasic dose dependency; Zn^2+^ supplementation might correct this.

Various estimates of the tolerable upper intake levels of Zn^2+^ for adults and children have been reviewed and discussed by Strand et al. (2017). Studies of the Lowest Observed Adverse Effect Level (LOAEL) for adults include 50–60 mg/day. Estimates of the No Observed Adverse Effect Level (NOAEL) for prolonged Zn^2+^ supplementation include 50 mg/day. The appropriate safety factor for side effects, such as interference with Cu^2+^ kinetics, is uncertain due to dietary differences. Various advisory bodies estimate the upper tolerable limit for prolonged supplementation in adults from 25 to 40 mg/day (reviewed by EFSA panel 2014; Strand et al. 2017); however, these estimates are controversial (reviewed in Nriagu 2007; Strand 2017; Freeland-Graves et al. 2020; Haile et al. 2021). Higher Zn^2+^ supplementation for the treatment of diseases over limited periods has been investigated in various studies of healthy and diseased human subjects. Administration of higher Zn^2+^ doses for treatment of type-2 diabetes and liver disease has been reviewed (Bloom et al 2021; Seyedmahalleh et al. 2023). In one study of human Zn^2+^ intake, 6 weeks of 150 mg/day caused reversible gastro-intestinal distress in half of healthy volunteers; however, other studies did not find similar distress from similar doses (reviewed by Haase et al. 2008, Plum et al. 2010). Six weeks is beyond the necessary period for improvement of COVID or long COVID with Zn^2+^ or metformin.

Many individuals safely self-administer simultaneous metformin and the 15–30 mg daily doses of Zn^2+^ that are present in ordinary multi-vitamins. The effects of metformin on body content of metals have not been extensively investigated. Co-administration of standard 1–2 g/day of metformin and 25 mg Zn^2+^/day (50 mg every other day) for 2 months increased serum Zn^2+^ and multiple anti-diabetic benefits as compared to metformin alone; no harm was reported (Younis et al. 2022). In type-2 diabetic subjects, metformin alone caused a slight increase in serum Zn^2+^ but not Cu^2+^ (Mohammad et al. 2021). However, serum or plasma Zn^2+^ is a small fraction of total body Zn^2+^ and is not a precise indicator of exchangeable intracellular Zn^2+^.

Likely benefits suggest trials of combined dosages of 1–2 g/day metformin and 50 mg/day Zn^2+^ for therapy against COVID-19 and long COVID. Supratherapeutic priming doses of metformin are not tolerable; however, a priming dose of Zn^2+^ above 50 mg/day over limited periods might provide faster onset of therapy with acceptable risk when combined with metformin. Based upon available human studies, daily Zn^2+^ dosage of 75 mg or higher for several weeks is unlikely to have unacceptable side effects; however, this must be evaluated in combination with metformin. At some undetermined dosage and dose duration, combined Zn^2+^- metformin might be harmful in individuals with compromised renal or hepatic function. The margin of safety is uncertain; however, the toxicity is slow in onset and can be detected by cautious monitoring. Pending better understanding, any doses of metformin and Zn^2+^ should be administered separately and not as the preformed complex. The metformin–Zn^2+^ complex is dissociable; therefore, dosage with 1–2 g/day of the preformed metformin–Zn^2+^ complex might deliver a dangerous amount of Zn^2+^. Individuals with pre-existing organ dysfunctions should be excluded from trials until risks are determined.

Zn^2+^ is not known to be a direct mutagen or carcinogen. However, epidemiological studies have reported that Zn^2+^ is increased or decreased in association with various forms of neoplasia (not reviewed here, see Ha et al. 2022). Dosage of 50 mg/day Zn^2+^ over 15 years had no effect on the incidence or growth of prostate cancer (Zhang et al. 2022). However, in the same study 75mg/day Zn^2+^ was reportedly associated with elevation of aggressive prostate cancer. In contrast, other studies found that prostate cancer is associated with Zn^2+^ deficiency. The ongoing controversy of safe long-term Zn^2+^ dosage is not relevant to short-term administration of elevated Zn^2+^ over several weeks. Investigations of any prophylactic or therapeutic combinations of Zn^2+^ and metformin must be preceded by determination of safety. However, the risks of COVID-19 and long COVID probably exceed the risk of combined metformin and Zn^2+^ over limited periods and perhaps longer.

## Summary and inferences

This paper discusses the chemical and biological basis for a relationship between three independent medical observations:

1. Zn^2+^ deficit is a risk factor for COVID-19; and oral Zn^2+^ supplementation has preventive and therapeutic actions against it.
2. Zn^2+^ deficit can also be a reversible cause of non-infectious (sterile) “inflammatory” tissue pathogenesis, e.g. skin disintegration, heart failure etc.
3. Metformin improves many of the same infectious and non-infectious inflammatory conditions that are improved by Zn^2+^ alone, including COVID-19.

Firstly, Zn^2+^ moderates the two SARS-CoV-2 proteases required for multiplication/invasion. Secondly, Zn^2+^ also moderates host proteases that execute both infectious and non-infectious inflammatory processes. Thirdly, theoretical and empirical chemistry agree that metformin forms Zn^2+^ complexes. Implications are twofold: (A) By analogy with other dissociable Zn^2+^ carriers, metformin coordination (Figs. 1-3) could increase absorption and body content of independent Zn^2+^ and its natural control of a protease network (Table 1). (B) Zn^2+^ coordination by biguanide derivatives can create protease inhibitors that act without elevation of basal cell Zn^2+^ concentration/content (Sweeney et al. 2003, Lockwood 2010, 2019). (A) is presently speculative; however (B) has been demonstrated (Fig. 4). By either or both mechanisms, metformin–Zn^2+^ interactions can suppress viral multiplication/invasion and the host inflammatory response while developing immunity clears the infection. In separate studies, metformin and Zn^2+^ have both been shown to improve COVID-19 outcomes by approximately 40%. Much background predicts that the combined effect of oral Zn^2+^ and metformin will prove to be greater than the independently observed effects of either agent alone. Even greater protection might be provided by addition of Paxlovid®.

COVID-19 is here to stay; and safe vaccines are regrettably met with societal distrust. Moreover, SARS-CoV-2 mutations to vaccine and drug resistance will require new anti-viral strategies (Yoon et al 2022). Resistance to Zn^2+^ interference with the reactions of two viral proteases and many host proteases is unlikely to arise. Independent of viruses, genetic variation in Zn^2+^ regulation and/or acquired Zn^2+^ deficiency could contribute to diverse non-infectious diseases, e.g. heart failure. Investigations of oral Zn^2+^ therapy alone and Zn^2+^-interactive drugs merit a high priority for prevention and therapy of diverse diseases.

Current treatment of severe COVID-19 involves inhibition of viral multiplication/invasion with anti-viral drugs and separate inhibition of the pathogenic host response with anti-inflammatory agents. In theory, the anti-proteolytic metformin–Zn^2+^ interaction can simultaneously treat both problems. However, clinical trials of the possible benefits of combined metformin and Zn^2+^ require no understanding or verification of the mechanisms proposed here. *Co-administration of metformin and Zn*^*2+*^* might save many lives with negligible cost while basic science progresses.*

## Appendix: Discovering or designing future biguanide moderators of the Zn^2+^-interactive protease web

Metformin might not be the optimal derivative for synergizing Zn^2+^ action against all hyper-catabolic conditions. Metformin might be best for safely decreasing low-grade inflammation over a lifetime, but not for prevention or treatment of a cytokine storm. Hundreds of derivatives of the biguanide metallophore have been identified; and many more are possible. Future biguanide derivatives can provide greater anti-proteolytic potency and efficacy. The benefit-risk equation for short-term administration preventing a potentially lethal condition differs from lifetime usage.

Inflammation can result from insufficient cell Zn^2+^ or excessive cell protease content or both; this question is fundamental to metal-interactive pharmacology. The possibility that metformin increases the exchangeable cell Zn^2+^ concentration is likely but not yet verified. However, the action of several biguanide derivatives against protease reactions has been conclusively demonstrated ex vivo (discussed in Lockwood 2019). Direct Zn^2+^-interactive protease inhibition might be required for intensive treatment of conditions involving a severe protease/anti-protease imbalance. The search for a Zn^2+^ carrier that increases the body Zn^2+^ content differs from the search for a metal complex that directly inhibits a protease reaction. Here, the search for direct protease inhibitors is discussed; and Zn^2+^ carriers are omitted although these two mechanisms might be simultaneously recruited.

Therapeutic biguanides might be found among known chemical structures or rationally designed a priori. Beyond the present scope, microwave-assisted combinatorial chemistry can produce a large number of biguanide derivatives to be identified (Mayer et al. 2004). Synthesis of many biguanides and biguanide-like compounds has been extensively reviewed by Grytsai et al. (2021a, b). The many possible derivatives can modify Zn^2+^ interactions with various protease active sites. The goal is to design or discover safe derivatives that bind to protease subsites so as align inhibitory Zn^2+^ or other metals with the catalytic thiolate-imidazole partners (see Lockwood 2019). Metformin, buformin, phenformin, proguanil, moroxydine, chlorhexidine and alexidine are metal-interactive biguanide derivatives that have been used in humans with differing side effects (reviewed by Kathuria et al. 2021). Phenformin, proguanil, metformin and chlorhexidine have been shown to be metal-interactive anti-proteolytic agents ex vivo (see above and Lockwood 2019). Many different moieties can be added at one or both termini to create a large number of different structures with different inhibitory specificities, potencies, exchange kinetics, and mammalian pharmacokinetics. Metformin has 10–100 fold less anti-proteolytic potency than phenformin in bioassay. The un-supplemented intracellular Zn^2+^ concentration of the perfused heart is sufficient to activate the anti-lysosomal effect of phenformin. This might be attributable to close phenformin mimicry of a protease substrate motif of proteases. Phenformin is a metal-interactive structural analogue of benzoyl arginine amide, which is an artificial substrate used in protease assays. Binding of the phenformin–Zn^2+^ complex to the active site of proteases aligns the inhibitory metal cation with the catalytic partners (illustrated in Lockwood 2019). Derivatives such as phenformin might require less metal concentration to inhibit proteases than metformin. Indeed, the anti-proteolytic action of phenformin might not be accompanied by any appreciable change in the independent cell Zn^2+^ concentration.

Guanylguanidine is metabolically inert; however, various derivative moieties can undergo metabolism. The derivative moieties of the biguanide metallophore determine pharmacokinetics, pharmacodynamics and off-target side effects. Metformin, phenformin, proguanil, and moroxydine can be absorbed from oral administration. However, chlorhexidine and alexidine are large molecules that do not readily cross membranes. Chlorhexidine and alexidine are used as topical antiseptics, including use against periodontal disease. However, metformin also improves periodontal disease by delivery from the circulation (Tseng 2022). Chlorhexidine is a non-absorbable structural analogue of proguanil (illustrated in Lockwood 2019); and both are metal-interactive anti-proteolytic agents. Proguanil is in widespread use against malaria despite toxicities. Chlorhexidine is used against inflammation from periodontal microbes only as a mouthwash. Thus, anti-proteolytic actions seem to underlie very different therapeutic benefits and risks of various biguanides.

Various types of chemical libraries contain millions of chemical structures, but do not account for metal interactions. Cheminformatic screening of libraries cannot retrieve “hand-in-glove” binding orientations of a drug within a known protease active site if a participating metal cation is omitted from the search criteria. Moreover, the basis of docking is the shape/charge complementarity of a drug and an enzyme active site. Additional short-range intermolecular attractive and repulsive forces can be involved in drug-protease interactions. Docking is a valuable tool; however, it does not use “first principles” of chemistry and cannot simulate the combination of attractive and repulsive forces in operation. A different approach is drug analogies with known substrate cleavage sites (discussed in Lockwood 2019). Biguanide derivatives can be rationally designed a priori and constructed to fit the combination of subsites surrounding the catalytic partners so as to align Zn^2+^ with the reaction mechanisms of proteases.

“Whole body” intervention in the Zn^2+^-responsive protease web offers great possibilities; however, it will require new thinking and much preliminary experimentation. Pharmaceutical engineers typically seek specific selective inhibitors of substrate binding to specific proteases that are known to be involved in a disease process, e.g. the viral main protease. However, the effect of a single inhibitor on a single protease reaction toward a single artificial substrate under maximized reaction conditions obviously does not characterize submaximal moderation of proteome degradation by the entire network of Table 1. Twenty amino acids and alternative splicing of 20,000 gene products create an immense number of cleavage sites with a range of protease affinities. Single agents obviously cannot decrease the binding of all substrates to all proteases of the inflammatory network. In various specialized cells individual web proteases might be differentially expressed; and the critical cell types, if any, have not yet been identified. The hierarchy of Zn^2+^ sensitivities among members of the network is not yet known.

Many questions remain to be investigated. Possible “trigger protease(s)” activating a transition from basal to hyper-catabolic function of the entire web have not been identified. If there are different triggers for reversible inflammation, and programed death (Eron et al. 2018), then treatment of some conditions might not require targeting of the entire Zn^2+^-responsive network. Serine protease reaction mechanisms are at least 10-fold less sensitive to Zn^2+^ than cysteine proteases*.* Caspases, calpains and cysteine cathepsins all have CysHis catalytic partners; however the Zn^2+^ sensitivity of the former two also involves allosteric exosites. Occupancy of allosteric sites and interference with reaction mechanisms are different inhibitory mechanisms of Zn^2+^. Zn^2+^ effects on membrane dynamics and autophagy are another consideration (citations above). For each protease a complete account requires consideration of (a) the natural inhibitory effects of Zn^2+^ and other metals alone, (b) the effect of the naked inhibitory drug in the absence of metals, and (c) the effect of the drug-metal complex. Combined administration of multiple protease moderators might be required for optimal efficacy against all members of the web. *A cocktail of non-toxic biguanide derivatives is not an unreasonable possibility*.

Problems continue with identification of the predominant biological effect for use in screening of biguanide candidates. In addition to moderation of protease reactions, metformin has many effects at various concentrations (not reviewed here). Screening for an inaccurate indicator of the primary mechanism might not identify the anti-inflammatory benefit. Metformin, buformin and phenformin were introduced into human use for their anti-hyperglycemic effect. The anti-diabetic metabolic actions of biguanides might differ from their anti-microbial and anti-inflammatory actions although they might be related. *Importantly, seeking the anti-hyperglycemic action of biguanides might not reveal an anti-viral or anti-inflammatory effect.* It is widely agreed that metformin has benefits “beyond control of glycemia”. Metformin can improve diabetic sequelae such as limb amputation and blindness despite modest or no improvement in glycemia and glycated hemoglobin. Conversely, testing the anti-microbial actions in cell culture will not reveal the effect of a drug on the host inflammatory/immune responses to infections in vivo. Identification of promising lead candidates for non-infectious anti-inflammatory action must be based upon metal-interactive moderation/ inhibition of protein degradation at relevant concentrations under conditions in mammalian tissues.

Phenformin was used at anti-hyperglycemic concentrations that are far above the anti-proteolytic concentrations that we measured. The anti-hyperglycemic action of phenformin requires concentrations that caused withdrawal of this drug due to myocardial effects in some individuals. The anti-hyperglycemic phenformin concentration risks weakening myocardial pumping action in individuals with a pre-existing vascular resistance, causing poor tissue perfusion, hypoxia, anerobic metabolism and lactic acidosis. However, the anti-proteolytic action of phenformin can be observed at much lower concentrations that might be tolerable under limited periods of administration. In our experience phenformin caused no functional compromise of the Langendorf perfused rat heart at effective anti-proteolytic concentrations of 10^-8^ M. Phenformin might not pose a significant cardiotoxic risk over limited periods at anti-proteolytic concentrations in most individuals under monitored administration (Lehrer 2020); however, this would have to be carefully investigated and compared to benefits.

The goal of pharmacological Zn^2+^ enhancement is to increase the occupancy time (or “interactive” time) of interfering Zn^2+^ between the catalytic partners (illustrated in Lockwood 2019). For prolonged administration, a biguanide derivative should provide safe reversible protease moderation but not irreversible denaturation. The actions of a reversible enzyme inhibitor depend upon drug concentration; however, the actions of a tight-binding, irreversible inhibitor depend upon the total drug dose relative to the total amount of titrated enzyme active sites. The sustained effects of a tight-binding inhibitor depend upon the relative rates of enzyme denaturation vs. replacement by de novo synthesis. Concentration-dependent effects of irreversible enzyme inhibitors are difficult to control even under intravenous administration.

Additional problems can be illustrated by our published experience with a non-selective protease such as cathepsin B and various biguanide derivatives against cleavage of artificial substrates. In the absence of metal cations, low phenformin concentrations inhibited approximately half of the endoprotease activity of cathepsin B toward an artificial peptide substrate (N-blocked to prevent exopeptidase action); however, much higher concentrations had no effect on the other half of the reaction. Inclusion of Zn^2+^ caused complete inhibition of the same reaction (Sweeney et al. 2003). Without Zn^2+^ phenformin functions as a competitive inhibitor of some substrate binding orientations but not others. Addition of Zn^2+^ changed the inhibition from competitive inhibition of substrate binding to inhibition of the reaction mechanism regardless of multiple possible substrate binding orientations.

Interpretive problems surrounding enzyme kinetics of non-selective proteases have not been reviewed or discussed. Ideal Michaelis-Menten reaction kinetics require one saturation constant (K_m_) one maximal reaction velocity (V_max_) and one inhibitor interaction with the substrate resulting from a single fit within the active site (K_i_). Firstly, a non-selective or broadly accepting protease can accept the same substrate in multiple binding orientations. This can result in multiple reaction velocities and multiple inhibitory constants against each binding orientation. Only a minority of the many proteases have absolute specificity for one bond of one substrate. Most proteases have kinetic preferences for some substrate cleavage sites but not absolute specificity (Ng et al. 2009). Cathepsin B is an example of a nearly non-specific endoprotease with exopeptidase activity (Schmitz et al. 2019). Secondly, larger synthetic peptide substrates can have multiple 3-D conformers, resulting in multiple interactions of the same substrate in different conformations. A competitive inhibitor might stop the reaction against some substrate binding orientations and conformations but have no effect on other binding orientations and conformations of the same amino acid sequence. Thirdly, certain biguanide-metal complexes can simultaneously compete with substrate binding while aligning inhibitory Zn^2+^ with the catalytic pair and inhibiting the reaction mechanism. Thus, some Zn^2+^-interactive drugs can combine competitive inhibition of substrate binding and non-competitive (but reversible) inhibition of the reaction mechanism, i.e. mixed kinetics. Fourthly, a dissociable metal complex can create multiple concentration-dependent variables, including the inhibitory effect of the naked drug, the inhibitory effect of the independent metal, and the inhibitory effect of the drug-metal complex.

Each of the multitude of cleavage sites of cell proteins has potentially different interactions with a non-specific protease and different kinetic properties. Accordingly, the same drug can exhibit different inhibitory kinetics against each cleavage site. Artificial substrates that are commonly used for protease assays are peptides that are N- blocked with a carbobenzyloxy moiety and conjugated with 7-amino-4-methylcoumarin (AMC) as a fluorophore, e.g. CBZ-PheArg-AMC or CBZ-ArgArg-AMC (discussed in Yoon et al 2022). Conceivable interactions of the blocking agent and fluorophore with the active site are unknown. The inhibitory action of biguanides against cathepsin B differed markedly against hydrolysis of CBZ-PheArg-AMC or CBZ-ArgArg-AMC substrate. At minimal therapeutic concentration of 10 μM metformin caused little inhibition of cathepsin B activity toward the CBZ-PheArg-AMC substrate either with or without Zn^2+^. However, sufficient metformin concentration caused partial inhibition of the hydrolysis of the CBZ-ArgArg-AMC substrate even without Zn^2+^. This is not surprising insofar as metformin might compete with the guanidines of the di-arginine substrate. Dibasic cleavage sites of many substrate proteins are well known, e.g. ArgArg, LysArg or LysLys. Interestingly, the SARS virus spike protein has a polybasic cleavage site including multiple arginines (Qiao et al. 2020, Whittaker 2021); this cleavage site is involved in invasion of host cells. A polybasic cleavage site on the spike protein is recognized by the host protease, furin. Furin is a target of anti-viral drug search (Wu et al. 2020). Zn^2+^-binding agents can inhibit furin (Podsiadlo et al. 2004). Recombinant human furin and viral spike protein are commercially available. It would be interesting to determine whether metformin or its Zn^2+^ complex might preferentially inhibit cleavage of the polybasic site of the viral spike protein by furin; (others are invited). The use of a reliable bioassay uniquely characterizes all protease-substrate interactions under the endogenous relationship between substrate concentrations, protease concentrations and Zn^2+^ concentrations.

In summary, the Zn^2+^-interactive protease network is a new concept. The effects of biguanides and Zn^2+^ on bioassayed bulk proteome turnover are consistent with protease reactions ex vivo; however, therapeutic intervention with future Zn^2+^-interactive biguanide derivatives requires much further development. Despite all uncertainties, guanylguanidine is a modifiable metallophore for enhancement of the natural action of Zn^2+^ and other metals against pathogenic hyper-catabolism of a web of proteases.

## Abbreviations

Metformin: 3-(Diaminomethylidene)-1,1-dimethylguanidine
Buformin: 2-Butyl-1-(diaminomethylidene)guanidine
Phenformin: 1-(Diaminomethylidene)-2-(2-phenylethyl)guanidine
Moroxydine: N-(diaminomethylidene)morpholine-4-carboximidamide
Proguanil: (1E)-1-[amino-(4-chloroanilino)methylidene]-2-propan-2-ylguanidine
Chlorhexidine: (1E)-2-[6-[[amino-[(E)-[amino-(4-chloroanilino)methylidene]amino]methylidene]amino]hexyl]-1-[amino-(4-chloroanilino)methylidene]guanidine
Alexidine: 1-[*N*′-[6-[[amino-[[*N*'-(2-ethylhexyl)carbamimidoyl]amino]methylidene]amino]hexyl]carbamimidoyl]-2-(2-ethylhexyl)guanidine
